# Gene Expression Correlations in Human Cancer Cell Lines Define Molecular Interaction Networks for Epithelial Phenotype

**DOI:** 10.1371/journal.pone.0099269

**Published:** 2014-06-18

**Authors:** Kurt W. Kohn, Barry M. Zeeberg, William C. Reinhold, Yves Pommier

**Affiliations:** Laboratory of Molecular Pharmacology, Center for Cancer Research, National Cancer Institute, Bethesda, Maryland, United States of America; Thomas Jefferson University, United States of America

## Abstract

Using gene expression data to enhance our knowledge of control networks relevant to cancer biology and therapy is a challenging but urgent task. Based on the premise that genes that are expressed together in a variety of cell types are likely to functions together, we derived mutually correlated genes that function together in various processes in epithelial-like tumor cells. Expression-correlated genes were derived from data for the NCI-60 human tumor cell lines, as well as data from the Broad Institute’s CCLE cell lines. NCI-60 cell lines that selectively expressed a mutually correlated subset of tight junction genes served as a signature for epithelial-like cancer cells. Those signature cell lines served as a seed to derive other correlated genes, many of which had various other epithelial-related functions. Literature survey yielded molecular interaction and function information about those genes, from which molecular interaction maps were assembled. Many of the genes had epithelial functions unrelated to tight junctions, demonstrating that new function categories were elicited. The most highly correlated genes were implicated in the following epithelial functions: interactions at tight junctions (CLDN7, CLDN4, CLDN3, MARVELD3, MARVELD2, TJP3, CGN, CRB3, LLGL2, EPCAM, LNX1); interactions at adherens junctions (CDH1, ADAP1, CAMSAP3); interactions at desmosomes (PPL, PKP3, JUP); transcription regulation of cell-cell junction complexes (GRHL1 and 2); epithelial RNA splicing regulators (ESRP1 and 2); epithelial vesicle traffic (RAB25, EPN3, GRHL2, EHF, ADAP1, MYO5B); epithelial Ca(+2) signaling (ATP2C2, S100A14, BSPRY); terminal differentiation of epithelial cells (OVOL1 and 2, ST14, PRSS8, SPINT1 and 2); maintenance of apico-basal polarity (RAB25, LLGL2, EPN3). The findings provide a foundation for future studies to elucidate the functions of regulatory networks specific to epithelial-like cancer cells and to probe for anti-cancer drug targets.

## Introduction

Progress in cancer biology and therapy depends in large part on comprehending the molecular interactions that govern key regulatory networks. The vast amount of data on gene expression in cancer cells should assist in reaching that goal, but effectively utilizing that information remains challenging. Most malignant solid tumors derive from epithelial tissues and retain epithelial characteristics to a variable degree that correlates inversely with malignant virulence. We aimed to utilize gene expression data for cell lines derived from various human tumors to elucidate molecular interaction networks controlling functions key to epithelial cell types, leading eventually to deeper understanding of the factors that govern transitions to mesenchymal character, a change that is thought to be central to acquisition of the ability of cancer cells to invade tissue and form distant metastases. The current work focuses on genes that are expressed selectively in epithelial cells, while a subsequent communication will focus on transitions between epithelial and mesenchymal cell states.

Epithelia are arguably the best defined as well as the embryonically earliest multicellular phenotype. A prominent characteristic essential to epithelia is tight junctions, which help to hold adjacent cells together and regulate transport of molecules through the paracellular space between adjacent cells [1]. Expression of a subset of genes that are associated with tight junctions may therefore serve as an indicator of epithelial character. This would be in accord with the general principle that genes that are expressed together in a variety of circumstances or cell types are likely to function together.

The relative expression levels of over 23,000 genes in the National Cancer Institute’s 60 human tumor cell lines (NCI-60) have been assembled into a freely and readily accessible database [2]. In a previous study, we showed that a set of mutually expression-correlated genes over the NCI-60 cell lines could be assembled into networks that control cell migration [3].

We now show that a subset of the NCI-60 cell lines that are selective in expression of certain tight junction-associated genes serve as a signature for epithelial character of tumor cells, and that genes positively correlated with that signature can be assembled into networks involved in the control of epithelial functions. We show that the expression patterns in the NCI-60 human cancer cell lines correlates well with expression in the CCLE/Broad cell lines.

Although gene expression at the mRNA level is not the sole determinant of corresponding protein expression (for which we do not yet have adequate data), the function correlations are striking.

The current work combines gene expression correlations with molecular interaction information directly from current scientific literature to assemble molecular interaction networks specific to epithelial-like cells. In addition to the bioinformatics analysis, an integral part of this study includes a comprehensive review of molecular interactions of genes (and gene products) having epithelial-related functions in human cancer cell lines.

## Methods

Gene expression profiles and correlations for NCI-60 human tumor cell lines were obtained using the “Gene transcript level z score” web-based tool provided by CellMiner (http://discover.nci.nih.gov/cellminer/). This tool provides relative quantitation for the cell lines from five microarray platforms [2]. CellMiner provided z-score correlations (r) of the expression of a given gene with respect to selectivity for the NCI-60 epithelial consensus (NEC) cell lines (see below and Table 1). Of the 22,379 genes for which there were validated data for the NEC genes in the CellMiner database, the fraction of genes having z-score (r) values greater than 0.90, 0.80, 0.70, 0.60, 0.50, 0.40 were respectively 0.0005, 0.0023, 0.0056, 0.013, 0.027, 0.059.

**Table 1:** Gene and cell line signatures of epithelial-like NCI-60 cell lines.

NEC Genes	Function	NEC Cell lines	Tissue of origin
CDH1/E-cadherin	AJ	MCF7	Breast
CLDN3	TJ	T47D	Breast
CLDN4	TJ	COLO205	Colon
CLDN7	TJ	HCC2998	Colon
MARVELD2/tricellulin	TJ	HCT116	Colon
MARVELD3	TJ	HCT15	Colon
OCLN/occludin	TJ	HT29	Colon
TJP3/ZO-3	TJ	KM12	Colon
		NCI_H322M	Lung
		OVCAR3	Ovary
		OVCAR4	Ovary

(NCI-60 epithelial consensus (NEC) genes and cell lines. See Fig. 4).

AJ, adherens junction; TJ, tight junction; NEC, NCI-60 epithelial consensus.

Gene expression data (CCLE_Expression_Entrez_2012-09-29.gct) for human tumor cell lines of the Cancer Cell Line Encyclopedia (CCLE) of the Broad Institute of MIT and Harvard were downloaded from (http://www.broadinstitute.org/ccle/data/browseData?conversationPropagation=begin). The downloaded file was pre-processed using a combination of UNIX commands and R programs, e.g., to remove entries for which the gene name was missing. The expression values for each gene were converted to a z-score across all samples in the dataset (i.e., mean zero and unit standard deviation), using the R program scale. The resulting matrix of gene expression profiles was saved as an R object. An in-house R package was used to compile and normalize the data from individual samples into a coherent dataset for each cancer type. The expression values for each gene were converted to a z-score across all samples in the dataset (i.e., mean zero and unit standard deviation), using the R program scale(). The resulting matrix of gene expression profiles was saved to hard drive as an R object.

Clustered image maps for gene expression and correlations were generated using an in-house R package.

**Figure 1:**
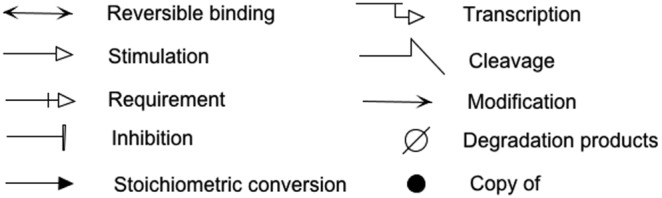
Molecular interaction symbols used in the MIM drawing tool [5]. A small filled circle (“node”) on an interaction line represents the entity or entities that are the consequence of the interaction. For example, a node on a binding interaction line represents the dimer or complex resulting from the binding; a node on a cleavage line represents the product(s) of the cleavage; a node on a modification line, represents the modified entity. For further description of the notation, see [4] and http://discover.nci.nih.gov/mim/mapDesc.html.

Information on molecular interactions and functions was assembled from recent literature in PubMed. The number of cited references was limited by citing recent publications that contain citations to earlier literature. The molecular interaction maps (MIMs) were prepared using the notation described by Kohn et al [4] and at http://discover.nci.nih.gov/mim/. The MIM symbols used in the current work are defined in Figure 1. The MIMs in the current work were constructed using the PathVisio-MIM software [5].

## Results and Discussion

### Expression Pattern of a Subset of Tight-junction and Adherens-junction Genes in the NCI-60 Cell Lines Defines a Candidate Epithelial Signature

Tight junctions are bands of specific structural proteins that seal cell-cell junctions and regulate passage of small ions or molecules through the intercellular space; they are an essential characteristic of epithelial cell types [6], [7]. The structural core of tight junctions is generally composed of one or more proteins from each of the following genes or gene families: TJP1-3, claudins (CLDN1-27), OCLN/occludin, MARVELD3, and MARVELD2/tricellulin [8]. We asked whether a subset of those tight-junction-family genes would exhibit an expression pattern of mutually correlated genes within the NCI-60 panel of human tumor cell lines. That pattern of selective expression could be a signature for epithelial character of human tumor cell lines in culture.

**Figure 2:**
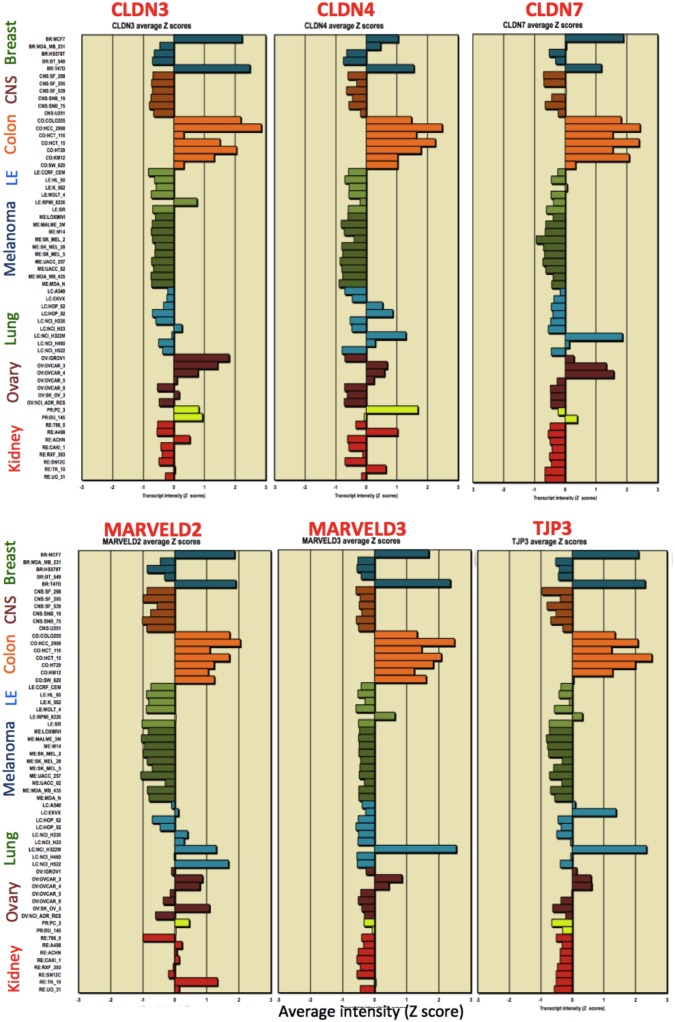
NCI-60 gene expression profiles for tight junction genes, showing a pattern of selective expression in 2 breast, 6 colon, 1 lung and 2 ovarian cancer cell lines. We refer to this subgroup of the NCI-60 cell lines as the “NCI-60 epithelial consensus” (NEC) cell lines. Genes selectively expressed by these cell lines are “NEC genes”.

Using the CellMiner NCI-60 analysis tools [2], we found that 7 members of the tight-junction-family genes formed a consensus pattern of mutually expression-correlated genes in 11 of the 60 NCI-60 cell lines. Figure 2 shows how closely those gene expression profiles resemble each other.

**Figure 3:**
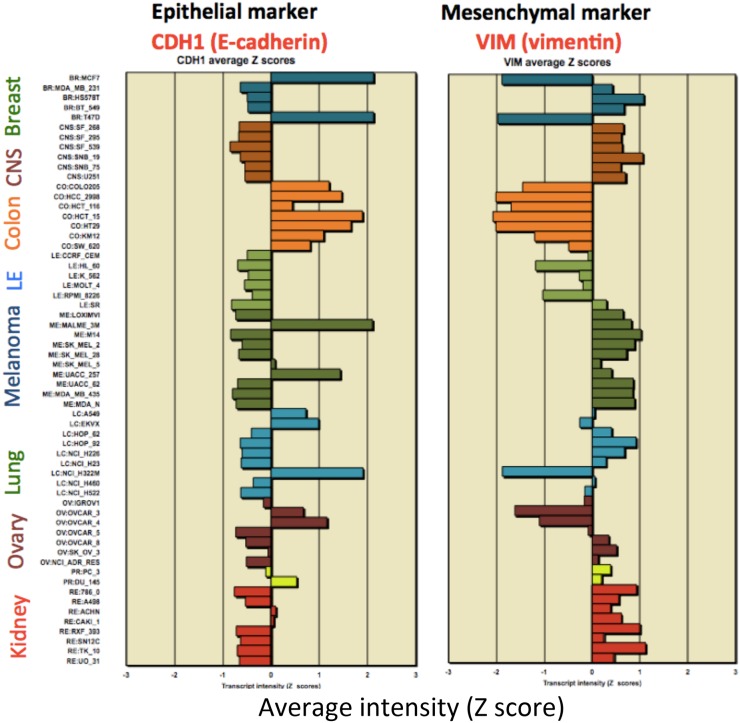
NCI-60 gene expression profiles for adherens junction gene CDH1/E-cadherin, an epithelial marker, and the mesechymal marker gene, VIM/vimentin. Nearly all of the cell lines that up-regulate CDH1 down-regulate VIM. Genes that are selectively not expressed by the NEC cell lines often have mesenchymal functions.

**Figure 4:**
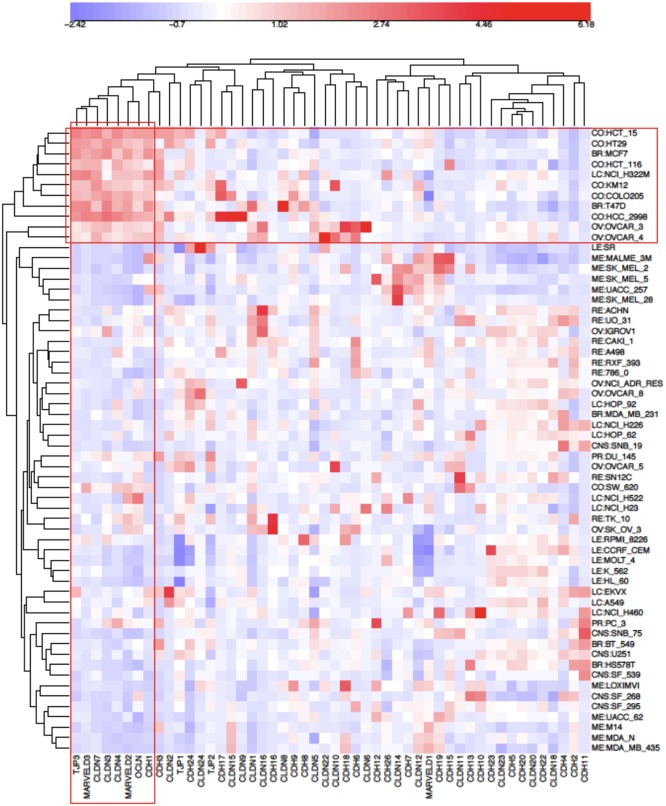
Clustered image map of NCI-60 mRNA expression levels for tight junction family and cadherin family genes. The genes and cell lines of the NCI-60 epithelial consensus (NEC) signature (Table 1) are boxed in red rectangles. The NEC genes are seen to constitute a subset of the tight junction and cadherin family genes.

Structurally associated with tight junctions are adherens junctions whose central component is the epithelial marker, CDH1/E-cadherin. Because of that functional relationship and the close similarity of its NCI-60 expression profile with that of the tight-junction genes displayed in Figure 2, we included CDH1 in an epithelial consensus signature (Figure 3, Table 1). The high mutual expression correlation of the genes listed in Table 1 is seen in Figure 4. Selective expression of those mutually correlated genes therefore was selected as a possible signature for epithelial character of human tumor cell lines in culture. We refer to those genes and the NCI-60 cell lines selectively expressing them as the “NCI-60 epithelial consensus (NEC)” signature. Although selective expression of NEC genes may be indicative of epithelial character, it may or may not indicate the presence of normal tight and adherens junction structures.

Figure 3 shows that the NCI-60 gene expression profile for CDH1/E-cadherin (and thus of the NEC genes in general) is nearly a mirror image of that of the mesenchymal marker gene, VIM/vimentin, suggesting that mesenchymal genes have selectively low expression in the NEC cell lines. The genes whose expression was highly selective in the NEC cell lines may be epithelial-like, and the genes whose expression was selectively low in the NEC cell lines may be mesenchymal or, more generally, non-epithelial.

The expression correlations of the NEC genes and cell lines in the context of all NCI-60 cell lines and all tight junction and cadherin gene family members is shown as a clustered image map (CIM) in Figure 4. We see that the NEC genes cluster is a subset of the tight junction and adherens junction gene families.

In addition to the NEC genes listed in Table 1, Figure 4 suggests that CDH3 (P-cadherin; correlation of gene expression with that of the NEC genes, r = 0.55) could be included in the cluster. CDH3 will be seen to co-cluster with the bone fide NEC genes in other data sets; therefore we consider CDH3 to be an ex-officio member of the NEC group.

Many of the gene family members included in the CIM do not have epithelial-related functions or are expressed at plasma membrane regions other than tight junctions. CDH2/N-cadherin, for example, forms adherens junctions in mesenchymal cells, which do not have cell-cell junctions of the type that is unique to epithelial cells [9]. Claudins (CLDNs) differ in their abilities to seal cell-cell junctions, and some of them form anion- or cation-specific channels in the narrow space between adjacent epithelial cells [10].

### Expression of Tight-junction and Cadherin Family Genes in the CCLE Human Tumor Cell Lines Derived from Epithelial Tissues

**Figure 5:**
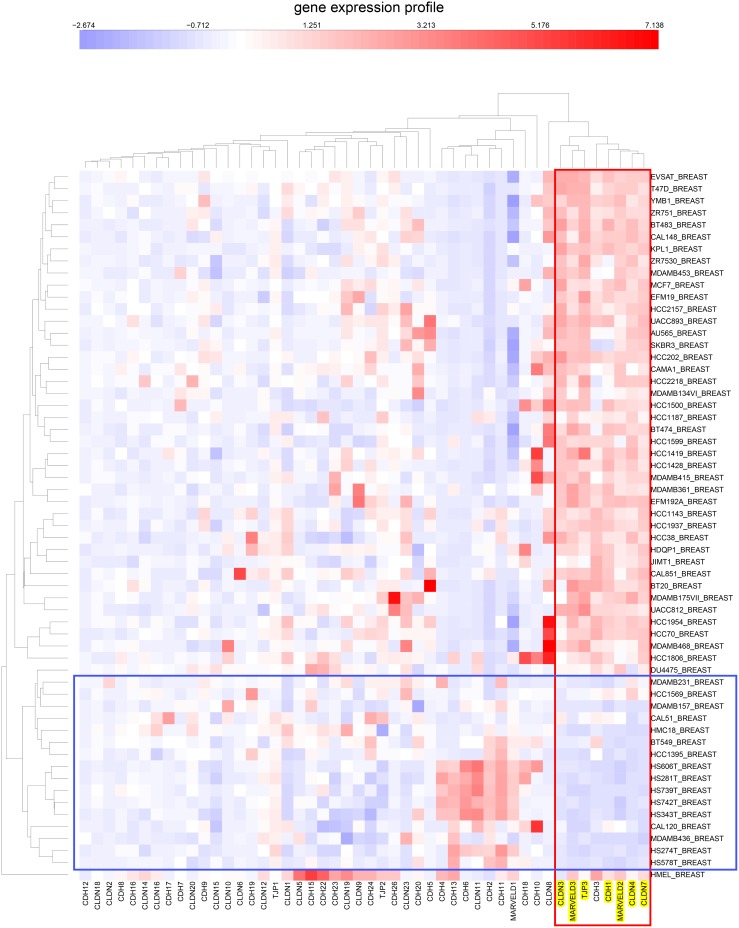
Clustered image map of the expression of tight-junction family and cadherin family genes (same gene set as in Figure 4) in CCLE breast cancer cell lines. The cluster containing the NEC genes is marked in a vertical box. The cell lines exhibiting distinctly reduced expression of NEC genes are enclosed in a horizontal box.

**Figure 6:**
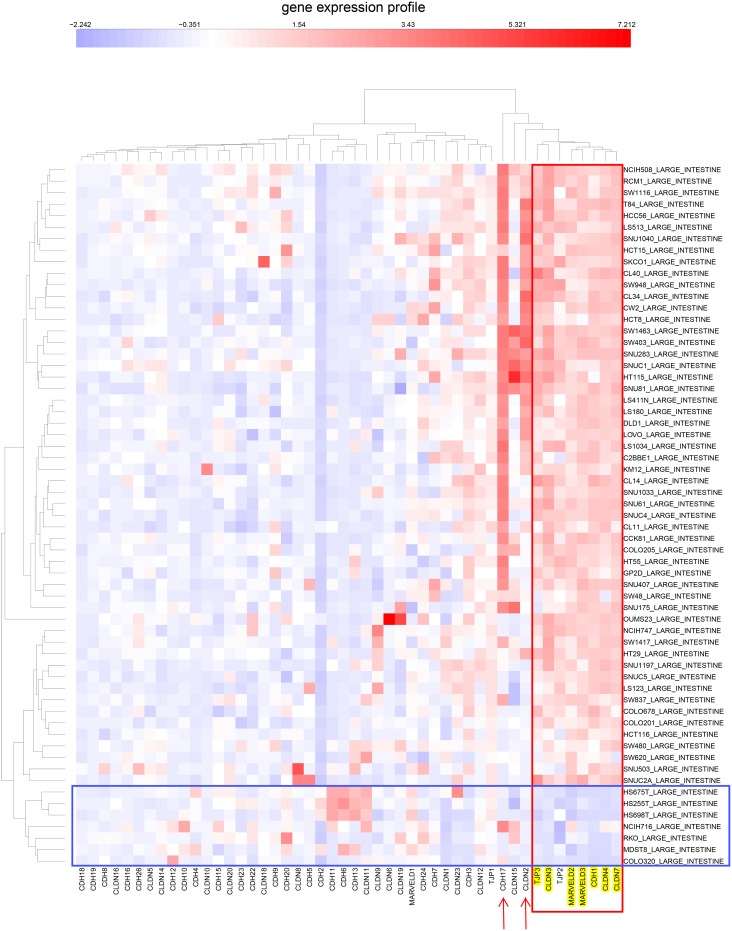
Similar to Figure 5, but for CCLE colon cancer cell lines.

**Figure 7:**
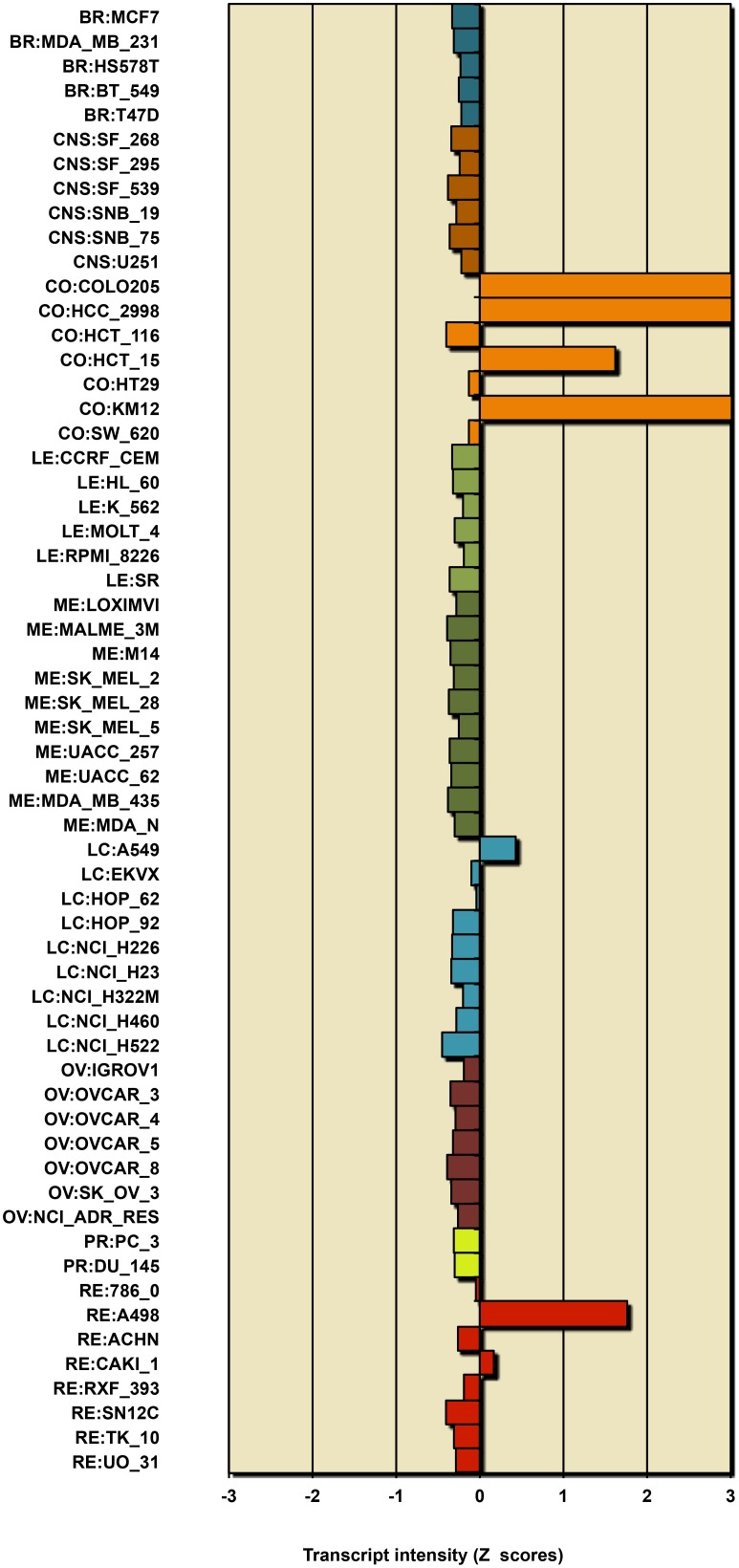
Expression of CDH17 mRNA in NCI-60 cell lines, showing selective expression in colon cancer cell lines.

**Figure 8:**
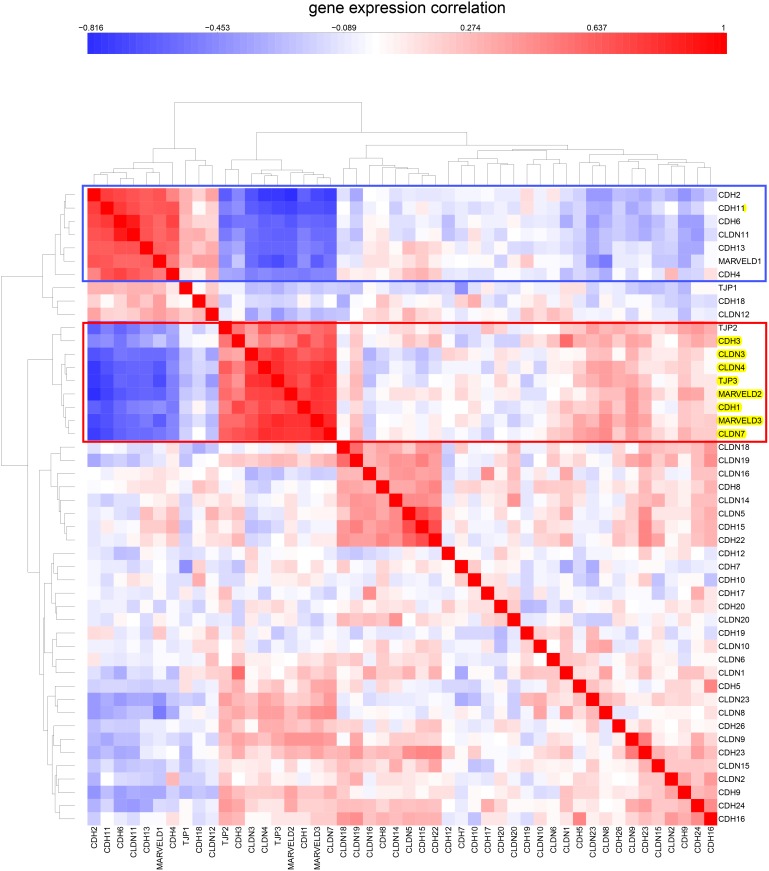
Expression correlations for tight-junction and cadherin family genes in CCLE breast cancer cell lines. The cluster containing the NEC genes is in a red box. A cluster containing genes whose expressions are inversely correlated relative to the NEC genes are in a blue box.

**Figure 9:**
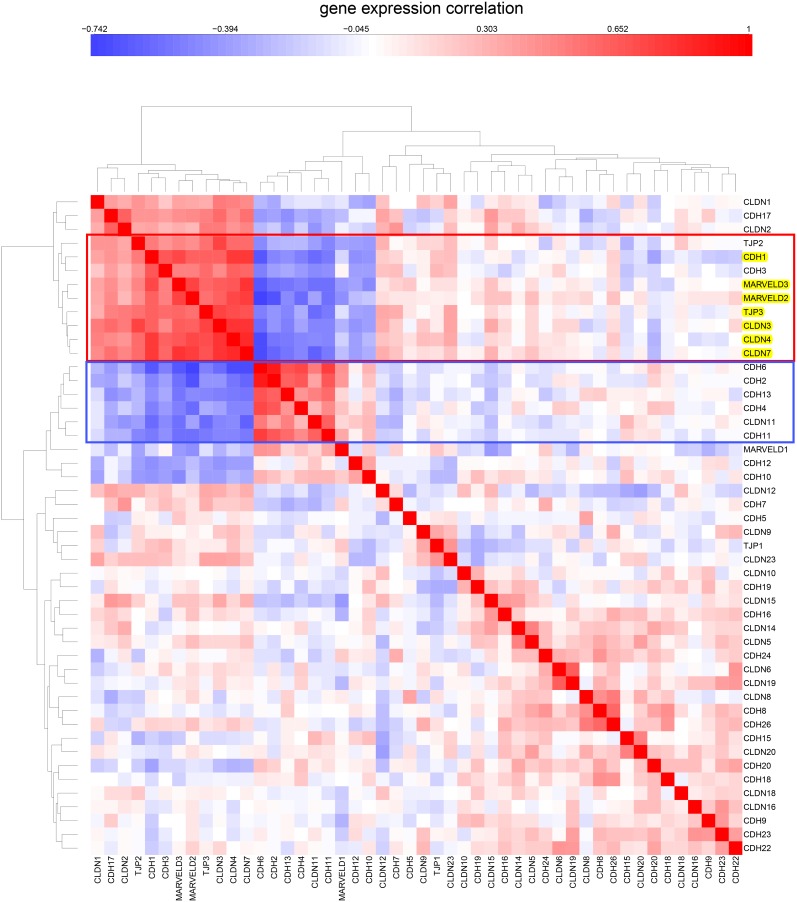
Similar to Figure 8, but showing CCLE colon cell lines.

Since the NCI-60 contain a limited number of cell lines per tissue type, we asked whether the NEC subset of tight-junction and cadherin family would also be evident in the data for the much larger number of human tumor cell lines of the Broad Institute’s Cancer Cell Line Encyclopedia (CCLE) [11]. Using the same set of genes that were used in the CIM of gene expression in the NCI-60 cell lines (Figure 4), we prepared CIMs of mRNA expression in CCLE breast and colon cell lines (Figures 5 and 6, respectively). These CIMs show that the NEC genes listed in Table 1 (except for OCLN, for which we found no data in CCLE) cluster together both in the CCLE breast and colon lines (Figures 5 and 6, red box), as they did in the CIM for the NCI-60 (Figure 4). In addition to the 7 NEC genes, those clusters included CDH3/P-cadherin in the breast lines and TJP2/ZO-2 in the colon lines. Figure 5 suggests that 16/58 (28%) of the CCLE breast cancer cell lines are non-epithelial. For the CCLE colon cancer cell lines (Figures 6 and 7), the corresponding fraction is 7/61 (11%). The expression correlations between gene pairs are shown in CIMs for CCLE breast and colon lines in Figures 8 and 9, respectively. For both the breast and the colon lines, the 7 NEC genes appear in a tight mutually correlated cluster (Figures 8 and 9, red box). These findings support the idea that the NEC genes serve as a signature for epithelial character in human tumor cell lines derived from epithelial tissues, and show minor variations of the composition of the NEC gene cluster in cancer cell lines from various tissues. In addition, both the breast and colon CIMs show a tight inversely correlated cluster (Figures 8 and 9, blue box), consisting of CDH2, CDH4, CDH6, CDH11, CDH13, and CLDN11, except that the breast cluster also contains MARVELD1. These genes thus tend to be down-regulated in the CCLE breast and colon cancer cell lines, and may function primarily in non-epithelial or mesenchymal cell types (which is well known for CDH2/N-cadherin). Thus the co-expressed NEC subset of tight-junction and cadherin family genes (Table 1) is also evident in the CCLE human tumor cell lines derived from epithelial tissues.

Most of the colon cell lines uniquely also express CDH17; selective expression of CDH17 is seen in 35 of the 54 (65%) epithelial-like CCLE colon lines (Figure 6). Among those 35 cell lines, 21 (60%) also express CLDN2; and among those 21 lines, 6 (29%) additionally express CLDN15 (Figure 6). Selective expression of CDH17 stands out as being specific to a large fraction of colon cell lines; this was also apparent in the NCI-60 gene expression profile where CDH17 was selectively expressed in 4 of the 7 colon cell lines (Figure 7). Thus colon cancer cells, and perhaps also colon cancers, may be stratified on the basis of expression of these genes. Expression of CDH17 in colon cancer cell lines, as well as colon cancer tissues, was previously reported by [12]. High expression of CDH17 was associated with reduced survival of colorectal cancer patients. CDH17 was found associated with beta1 integrin and other factors suggesting effects on cell adhesion and extracellular matrix interactions. In the CCLE ovarian cancer cell lines, approximately half of the lines showed high expression of CLDN16, although the distinction between epithelial-like and mesenchymal-like cell lines was not clear.

### The Cell Lines of the NCI-60 Epithelial Consensus (NEC) Serve as a Seed to Discover other Genes that are Selectively Expressed by these Cell Lines and that have Epithelial-specific Functions

Many tight junction family genes also function elsewhere in the cell, and particular NEC cell lines may or may not have normal tight junctions. We ask however whether other genes selectively expressed in the NEC cell lines have additional epithelial-related functions, which would further test the inference that NEC gene expression provides a signature for epithelial character of tumor cells.

Having defined an NEC cell line signature based on selective expression of a subset of tight junction and cadherin family genes, we used the pattern comparison tool of CellMiner to identify other genes selectively expressed (or selectively not expressed) by those NEC cell lines. We found 76 genes whose z-score correlations with respect to selective expression in the NEC cell lines was r>0.75. For each of those 76 genes, we assembled information about molecular interactions and functions from recent scientific literature. We found relevant information for 44 of the genes (“NEC-correlated epithelial genes”; Table 2); the remaining 32 had no published information linking them to epithelial-specific functions (Table 3), but these genes likely have functions in epithelial tumor cell lines that remain to be discovered. Genes that exhibited the strongest negative correlations with respect to selective expression in the NEC cell lines (“NEC-anti-correlated genes”) are listed in Table 4. Those genes may have non-epithelial or mesenchymal functions.

**Table 2:** All genes highly correlated (r>0.75) with selective expression in NCI-60 epithelial consensus (NEC) cell lines and having known epithelial functions.

Gene	Alternate name	Correlation (r)	Function in epithelia
ESRP1		0.971	RNA splicing
MARVELD3		0.949	Tight junction
RAB25		0.943	Cell polarity
IRF6		0.928	Terminal differentiation
CLDN7	claudin 7	0.927	Tight junction
ATP2C2		0.897	Calcium signaling
GRHL2		0.891	Cell polarity
ST14	matriptase	0.890	Terminal differentiation
S100A14		0.885	Calcium signaling
MAPK15	ERK8	0.883	Regulates estrogen receptor
SPINT1		0.883	Terminal differentiation
ELMO3		0.878	Intestinal villi
BSPRY		0.871	Calcium signaling
TJP3	ZO-3	0.868	Tight junction
CBLC		0.861	Intestinal villi
PLEKHG6		0.854	Cell polarity
ESRP2		0.853	RNA splicing
EPCAM		0.835	Tight junction
PRSS8		0.834	Terminal differentiation
OVOL1		0.834	Terminal differentiation
EPN3	epsin 3	0.831	Cell polarity
SCNN1A		0.827	Sodium channel
GRHL1		0.826	Cell polarity
ADAP1	CENTA1	0.821	Cell polarity
EPHA1		0.817	Cell-cell junction
CRB3	Crum3	0.808	Tight junction
ANXA9	annexinA9	0.807	Desmosomes
IL17RE		0.806	Epithelial immune pathway
CGN	cingulin	0.802	Tight junction
EHF	ESE3	0.802	Progenitor cell differentiation
CLDN4	claudin 4	0.802	Tight junction
LLGL2		0.797	Cell polarity
MACC1		0.796	Metastasis competency
MYO5B		0.785	Cell polarity
LNX1		0.780	Tight junction
MARVELD2	tricellulin	0.773	Tight junction
CDH1	E-cadherin	0.772	Adherens junction
CLDN3	claudin 3	0.760	Tight junction
PPL	periplakin	0.758	Desmosomes
RNF43		0.756	Colon selective; beta-catenin path
CAMSAP3	KIAA1543, Nesha	0.755	Adherens junction
OVOL2		0.754	Terminal differentiation
ELL3		0.753	Proliferation/differentiation
AP1M2	AP-1 subunit	0.749	Cell polarity

**Table 3:** All genes highly correlated (r>0.75) with selective expression in NEC cell lines but having no reported epithelial functions.

Gene	Alternate name	Correlation (r)
C1orf172		0.947
C1orf210		0.929
PRRG2		0.907
CCDC64B		0.906
PPP1R14D		0.896
TMC4		0.887
TMEM125		0.885
ANKRD56		0.873
CNKSR1		0.868
CDC42BPG		0.865
ILDR1		0.862
FAM83B		0.842
LAD1		0.832
CDS1		0.830
PRSS22		0.829
GRTP1		0.820
B3GNT3	TMEM3	0.813
ARHGEF16	EPHEXIN4	0.810
ZNF165		0.804
MPZL3		0.797
GOLT1A	GOT1	0.792
MPZL2	EVA1	0.790
C17orf28		0.783
SLC37A1		0.778
TBC1D30		0.776
KRTCAP3	KCP3	0.776
MAPK13		0.775
SULT2B1		0.769
C11orf52		0.768
C9orf152		0.760
GYLTL1B		0.758
PRR15L		0.751

NEC, NCI-60 epithelial consensus.

**Table 4:** All genes negatively correlated (r<–0.6) with respect to selective expression in NEC cell lines.

Gene	Alternate name	Correlation (r)
LIX1L		–0.862
VIM	Vimentin	–0.858
MSN	Moesin	–0.761
CCDC88A	Girdin	–0.747
EMP3	YMP, HNMP1	–0.730
CMTM3	BNAS2	–0.724
QKI	QK3, Quaking	–0.705
FKSG43		–0.705
IKBIP	IKIP	–0.700
AP1M1	CLAMPM2	–0.676
BICD2	Bicaudal	–0.670
LEPRE1	Leprecan	–0.665
CHST10	HNK1ST	–0.665
STARD9		–0.664
GNB4		–0.660
FAM126A		–0.647
SYDE1		–0.644
LRP12		–0.642
MAP7D1		–0.640
SACS	Sacsin	–0.639
NR3C1		–0.626
RECK		–0.625
DYRK3		–0.620
MAP7D3		–0.610
ST3GAL3		–0.609
SOAT1		–0.608
SLC35B4		–0.605
SPG20	Spartin	–0.596
ELOVL5		–0.595
ETS1		–0.595

NEC, NCI-60 epithelial consensus.

### Gene Expression Dichotomy between Epithelial-like and Mesenchymal-like Cell Lines

**Figure 10:**
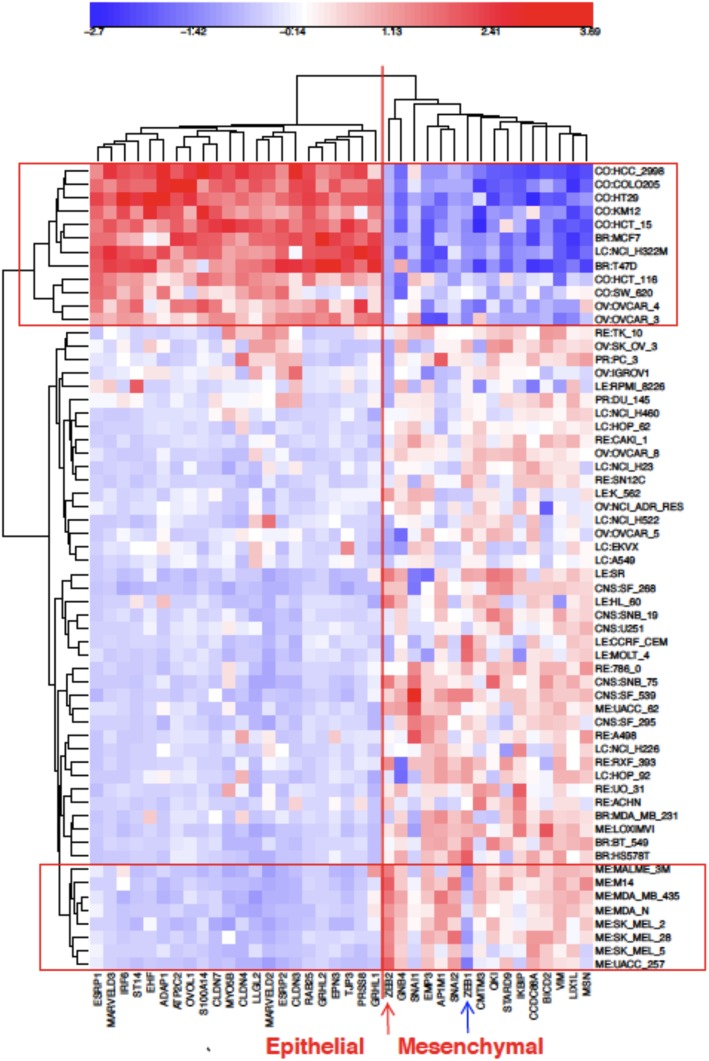
Expression of epithelial and mesenchymal genes in the NCI-60 cell lines. The genes shown were the most up-regulated (“epithelial”) or down-regulated (“mesenchymal”) in the NEC cell lines from Tables 2 and 4, respectively. The NEC cell lines cluster together as expected (top rectangle). Melanoma cell lines formed a separate cluster (bottom rectangle). Note the high expression of ZEB2 and low expression of ZEB1 (red and blue arrows at bottom).

NEC-correlated epithelial genes from Table 2 and NEC-anti-correlated genes from Table 4 were combined in a clustered image map (CIM) of mRNA expression in the NCI-60 cell lines (Figure 10). As expected, they show a sharp dichotomy between epithelial-like and non-epithelial cell lines with the NEC cell lines in a tight cluster (upper red box). Interestingly, 8 of the 9 melanoma cell lines cluster together (bottom red box), suggesting that gene expression patterns in these cell types differs from other non-epithelial cell types. Particularly notable in these melanoma cell lines is that they tend to express ZEB2 selectively, but not ZEB1 (arrows at bottom of the CIM).

**Figure 11:**
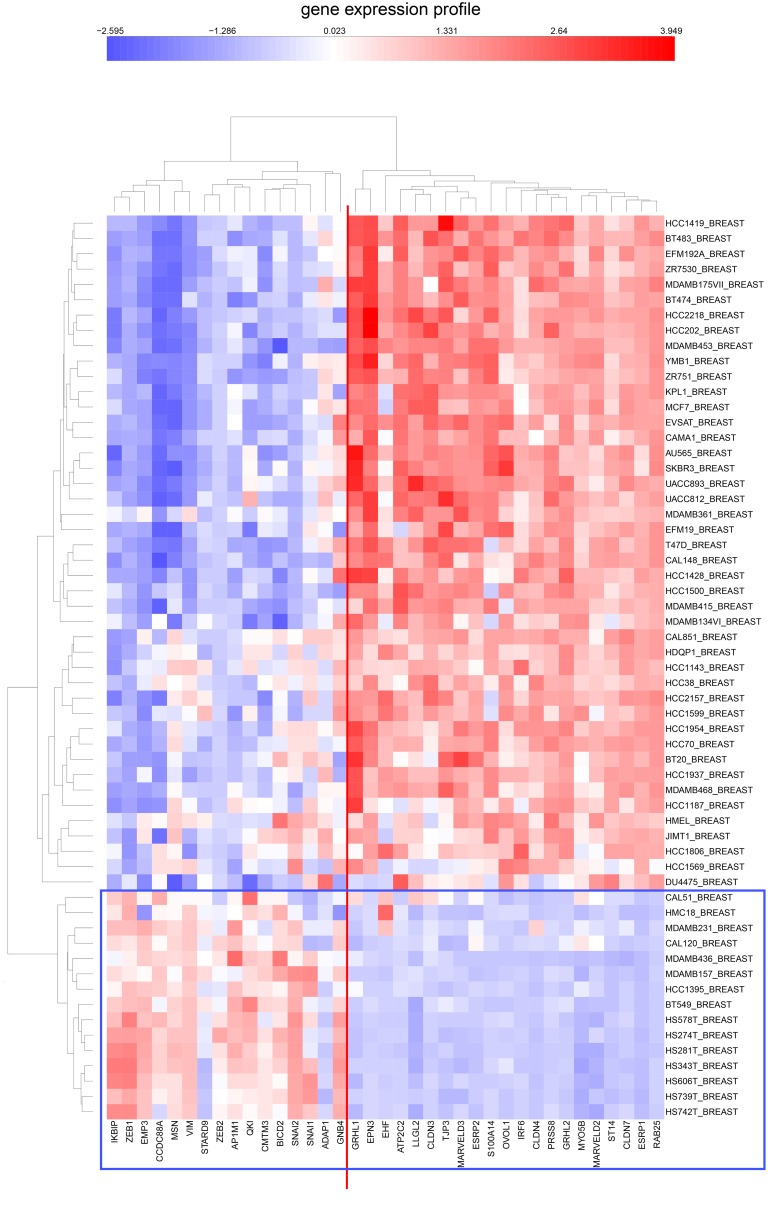
Expression of epithelial and mesenchymal genes in CCLE breast cancer cell lines. The gene set was the same as in Figure 9, except that there were no data for LIXL1. The clustering of the epithelial and mesenchymal genes was the same in the CCLE breast cancer cell lines as in the NCI-60 cell lines.

**Figure 12:**
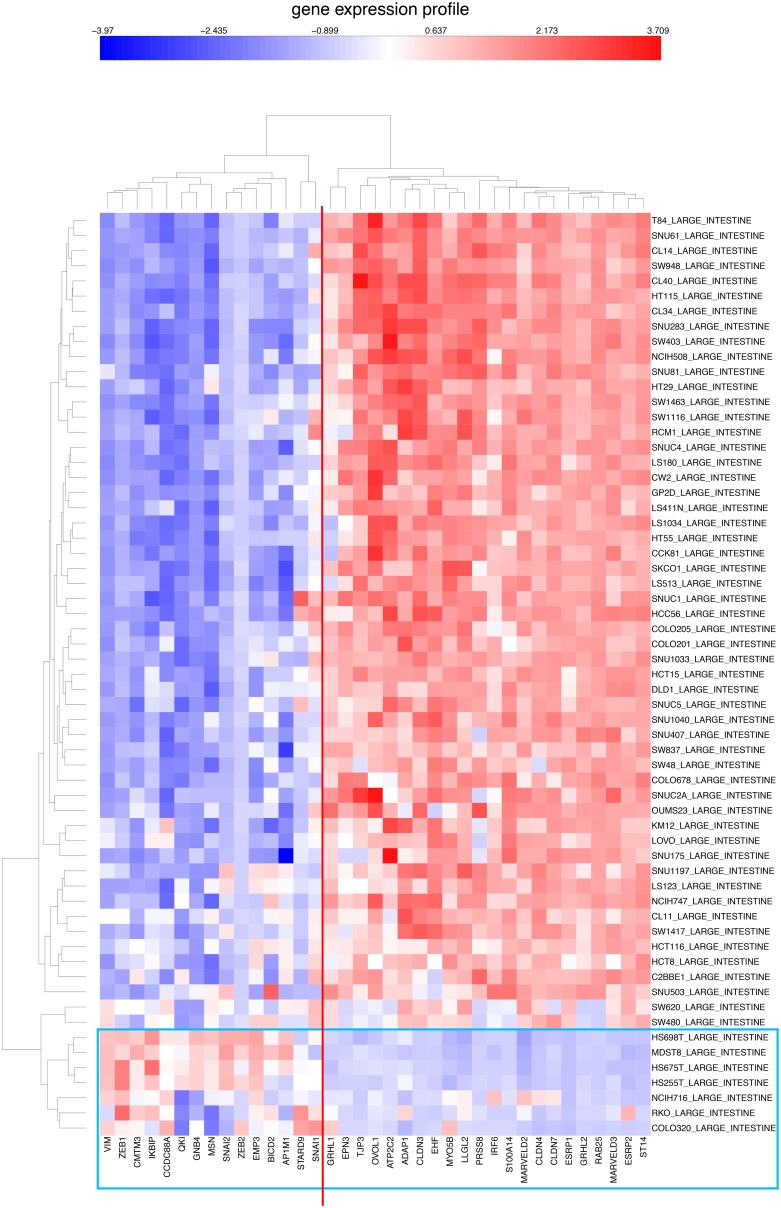
Similar to Figure 11, but for CCLE colon cancer cell lines.

**Figure 13:**
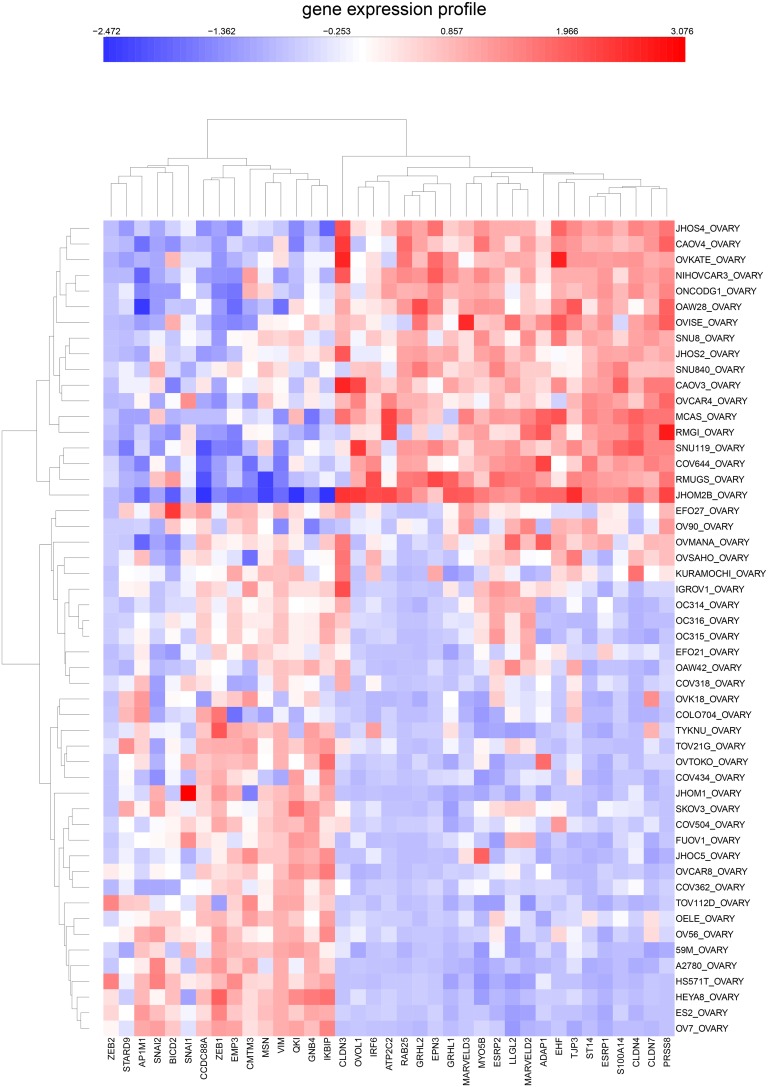
Similar to Figure 11, but for CCLE ovarian cancer cell lines.

We then asked whether this gene expression dichotomy, which was based on NCI-60 data, would hold up in CCLE cell lines. We found that this is clearly true for CCLE cell lines derived from breast, colon, and ovary (Figures 11–13). Moreover, these mRNA expression CIMs allow us to estimate the fraction of the cell lines from each tissue type that have a non-epithelial or mesenchymal gene expression pattern (Table 5). The values for the breast and colon lines were very close to those that were based on expression of tight-junction and adherens junction genes (Figures 5 and 6, Table 5). The CCLE ovarian cancer cell lines had a relatively large percentage of non-epithelial or mesenchymal-like cell lines (65%, Figure 13), perhaps due to the large incidence of ovarian tumors of mesothelial origin, which perhaps have a non-epithelial gene expression profile (the non-epithelial cluster divides into two sub-clusters that perhaps distinguish between predominantly mesenchymal versus mesothelial character).

**Table 5:** Number of epithelial and mesenchymal (or other non-epithelial) cell lines in CCLE human cancer cell line sets from various tissues.

Tissue	Gene set	Mesench/Epithelial	% mesenchymal	Figure
breast	TJ/CDH (a)	16/41 (c)	28.1%	5
breast	epith + mesench (b)	15/43	25.9%	11
colon	TJ/CDH (a)	7/54	11.5%	6
colon	epith + mesench (b)	7/52 (d)	11.9%	12
ovary	epith + mesench (b)	34/18	65.4%	13
pancreas	epith + mesench (b)	11/31 (d)	26.2%	–
stomach	epith + mesench (b)	13/24 (c)	35.1%	–

(a) Tight junction plus cadherin family genes.

(b) Epithelial and non-epithelial or mesenchmal genes from Tables 2 and 4, respectively.

(c) One intermediate cell line not counted.

(d) Two intermediate cell line not counted.

The gene sets in Tables 2 and 4 thus distinguish epithelial-like from mesenchymal-like character in human tumor cell lines. The next question is whether those gene sets participate in a coherent functional network. In the current work we address that question for the epithelial-related genes in Table 2, as well as interacting genes whose NEC correlation is significant, although not high enough to meet the criteria for inclusion in Table 2.

In the following description of the molecular interactions of NEC-correlated genes, the first occurrence of a gene name in each section is shown in bold, along with the correlation value (r) for selective expression in the NEC lines, as given by CellMiner.

### Molecular Interactions and Signaling at Cell-cell Junctions of Epithelial-like Cancer Cells

**Figure 14:**
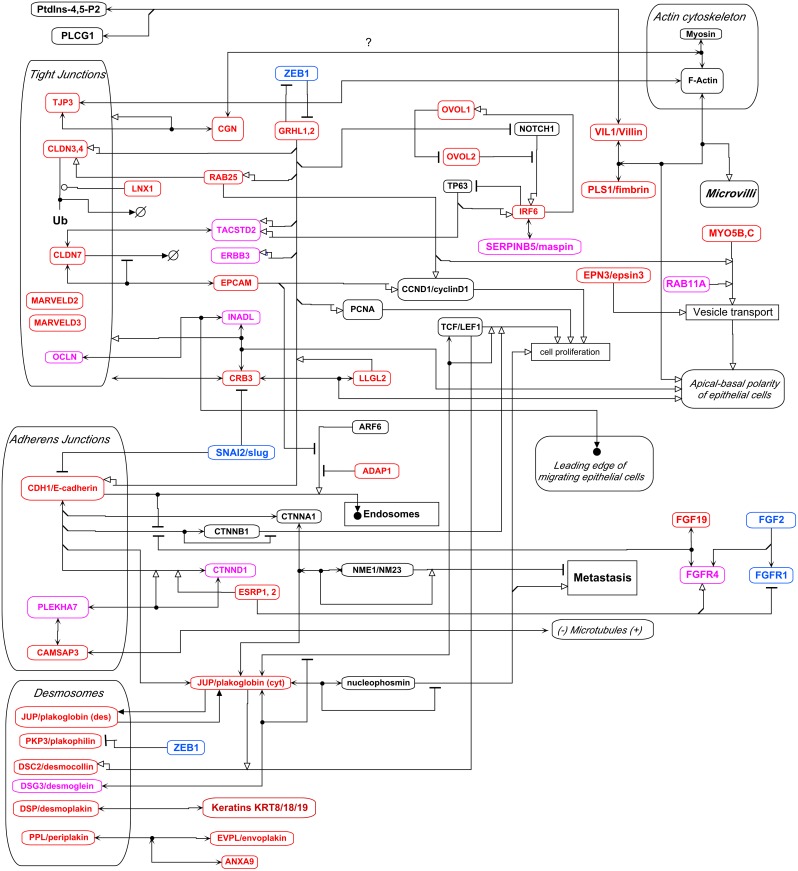
Molecular interaction map (MIM) of interactions at cell-cell junction complexes. Genes selectively expressed in the NCI-60 epithelial consensus (NEC) cell lines are shown in red. Symbol definitions are shown in Fig. 1.

Many of the most highly NEC-correlated genes were found to interact in a molecular interaction network related to cell-cell junctions: tight junctions, adherens junctions, and desmosomes; these genes are colored red in the molecular interaction map (MIM) in Figure 14). The relevant network interactions and functions of the NEC-correlated genes implicated in these functions are described below. At its first occurrence in each section, the name of each of those genes is in bold type along with its NEC expression correlation (r).

### Interactions at Tight Junctions

The central components of tight junctions include members of the claudin family of genes, which encode tetra-spanning transmembrane proteins that associate laterally to form circumferential anastomosing bands near the apical region of epithelial cells. Their extracellular domains, which associate intercellularly in the space between adjacent cells, regulate ionic paracellular permeability between apical and basolateral regions of the extracellular space, and allow ion permeation with selectivity that differs among different claudins [13] [14]}[6]. The claudins whose expressions correlated most closely with the NEC cell line pattern were **CLDN3, 4 and 7** (r = 0.76, 0.80 and 0.93, respectively) (Figure 4). Note that the expression pattern for **CLDN7** (r = 0.93) is a nearly perfect match to the NEC pattern. Closely associated with the claudins in tight junctions is **OCLN**/occludin (r = 0.58), although its precise role in tight junctions is not clear. When epithelial cells migrate during wound healing, OCLN in complex with **INADL** (r = 0.69) moves from cell-cell junctions to the leading edge of the migrating cells [15]. Also included in the tight junction structure are **MARVELD3** (r = 0.95), a tetraspanning transmembrane protein [8] and **MARVELD2**/tricellulin (r = 0.77), which is localized at 3-cell junctions in the epithelial monolayer [10], [14]. Note that MARVELD3, like CLDN7, exhibited a nearly perfect match to the NEC pattern (Figure 2). Tight junction structures include members of the TJP/zona occludens family, of which only **TJP3**/ZO-3 (r = 0.87) correlated strongly with the NEC gene expression pattern (Figure 4).

TJP proteins link tight junctions with the cortical actin cytoskeleton and are required for its structural integrity [16]–[18]. Possibly also involved is **CGN**/cingulin (r = 0.80), which can bind both TJP1–3 and actomyosin [19], [20] (Figure 14). The TJP involvement may differ among cell types. We find TJP3 most prominently correlated with the expression of other NEC genes. In the CCLE colon cancer cell lines, however, TJP2 correlated in the same cluster with the NEC genes (Figure 6), and TJP1 appeared in the NEC-correlated gene cluster in the CCLE ovarian cancer cell lines. Thus, while TJP3 was selectively expressed in epithelial-like cancer cell lines, TJP1 and 2, which are known also to participate in tight junction structures, may have more general functions in most of those cell lines.

Directly associated with tight junctions are **CRB3**/Crum3 (r = 0.81) and **INADL**/Patj (r = 0.69) (Figure 14), which bind to each other and are part of a complex that maintains apical/basolateral polarity of epithelial cells [21], [22]. This complex is down-regulated upon epithelial-mesenchymal transition [23]. CRB3 also binds **LLGL2** (r = 0.80), which participates in the complex that maintains apical/basolateral polarity. LLGL2 was able to reverse an epithelial-mesenchymal transition [24].

Tight junctions are also affected by the trans-membrane glycoproteins **EPCAM**/TACSTD1/TROP1 (r = 0.84) and **TACSTD2**/TROP2 (r = 0.64), both of which bind CLDN7 (Figure 14). In the absence of EPCAM, CLDN7 protein (but not its mRNA) is depleted and the barrier function of tight junctions is impaired [25]. Unlike EPCAM, which is expressed in various epithelia, TACSTD2 is expressed in stratified epithelia, but not in colonic or other simple epithelia [26].

EPCAM binds CLDN7 tightly and inhibits its degradation, but does not localize at tight junctions. Instead it localizes at lateral cell-cell junctions, where it sequesters CLDN7 in regions distinct from tight junctions [27]. Although localized similarly to adherens junctions, EPCAM does not bind CDH1/E-cadherin. These actions of EPCAM impair tight junctions and promote metastasis [25], [27]. This unusual circumstance of an NEC-correlated gene associated with perturbation of epithelial cell-cell junction structures suggests a possible abnormality of epithelial cancer cell lines in culture, which however remains to be tested in normal epithelial cells. One possibility is that EPCAM is associated with epithelial cell proliferation during wound healing, and that epithelial cancer cell lines in culture proliferate as in wound healing, thus explaining the highly NEC-correlated EPCAM expression. Consistent with this possibility, EPCAM induces transcription of cyclin D1; in the absence of EPCAM, cyclin D1, phosphorylated-Rb and cell cycle progression are suppressed [28].

The highly NEC-correlated gene **LNX1**/PDZRN2/MPDZ (r = 0.78) codes for a PDZ domain-containing E3 ubiquitin ligase that targets CLDN3, as well as serine/threonine kinase PBK and other proteins [29]. LNX1-mediated ubiquitination and degradation of PBK inhibited cell proliferation and enhanced cell sensitivity to doxorubicin [29]. LNX1 may have a role in tight junction organization or turnover through its association with CLDN1, CLDN3 and TJP1 [30], [31]. The manner in which the actions of LNX1 are functionally integrated however remain to be elucidated.

### Interactions at Adherens Junctions

Closely associated with tight junctions are adherens junctions, of which **CDH1**/E-cadherin (r = 0.77) is the major structural component. Additional components of adherens junctions are **CAMSAP3** (r = 0.76) and **PLEKHA7** (r = 0.52), which bind to each other in a complex that could bring together several components: CAMSAP3 binds the minus ends of microtubules, and PLEKHA7 binds **CTNND1**/p120-catenin (r = 0.48), which in turn binds CDH1/E-cadherin [20], [32], [33] (Figure 14). The complex formed by these bindings links adherens junctions to microtubules. PLEKHA7 also binds CGNL1/paracingulin, which binds CGN (possibly indirectly) [20], thereby potentially linking between adherens junctions and tight junctions. Such linkage however would not be effective in the NEC cell lines, because these plastic-grown cells did not express CGNL1. Adherens junctions are disassembled when CDH1/E-cadherin is taken into endosomes, an action that is promoted by ARF6 and inhibited by **ADAP1**/CENTA1 (r = 0.82). Thus ADAP1, whose expression is highly NEC-correlated, maintains adherens junctions and preserves epithelial character [34], [35] (Figure 14).

### Interactions at Desmosomes

Desmosomes confer strong cell-cell adhesion in association with adherens junctions in epithelial cells and provide linkage to the cytoskeleton, particularly keratin intermediate filaments. The interactions of the desmosomal proteins are shown in Figure 14. Desmocollins, such as **DSC2** (r = 0.60), and desmogleins, such as **DSG3** (r = 0.38), are desmosomal cadherins that form calcium-dependent cell-cell junctions similar to those of the adherens junctions of CDH1/E-cadherin. Plakophilins, such as **PKP3**/plakophilin (r = 0.71), and **JUP**/plakoglobin (r = 0.70), constitute the outer dense plaque that connects to the desmosomal cadherins and to the cytoskeletal linker protein **DSP**/desmoplakin (r = 0.62) on the cytoplasmic side of the plasma membrane (Figure 14). PKP3 is transcriptionally repressed by **ZEB1**, an NEC-negatively correlated gene (r = –0.58), thereby loosening epithelial cell-cell adhesion and promoting cell invasion and metastasis [36]. In stratified epithelia, desmoplakin links desmosomes to intermediate filaments. Another desmosome component, **PPL**/periplakin (r = 0.76), binds **EVPL**/envoplakin (r = 0.49) and the two bind intermediate filaments [37]. PPL associates with **ANXA9**/annexinA9 (r = 0.81); the two proteins, whose expression is highly correlated with the NEC genes, co-localize at cell-cell junctions of epithelial cells [38]. ANXA9 has been reported to be up-regulated in prostate and colon cancers [39], but details of its function have not been elucidated. The PPL-EVPL dimer associates with desmosomes via the N-terminal region of PPL [40]. Thus the mRNA expressions of 5 of the 7 above-mentioned epithelial desmosomal proteins correlated strongly (r>0.60) with the NEC gene expression pattern (Figure 14), while the remaining 2 correlated at lower but yet significant levels. Figure 14 shows how desmosomal proteins contribute to epithelial-specific interactions, several of which are mediated by JUP/plakaglobin.

**JUP**/plakoglobin (r = 0.70) is a structural component of both desmosomes and adherens junctions, where it binds the cytoplasmic tail of CDH1/E-cadherin [36], [41] (Figure 14). Plakoglobin helps maintain epithelial character and low proliferation rate. It binds to and inhibits the pro-mesenchymal actions of nucleophosmin (Figure 14), and may thereby convert mesenchymal cells to an epithelial-like state with low proliferation rate and reduced invasiveness [42]. In conjunction with TCF/LEF, plakoglobin functions as a transcription factor, which can act as a switch to induce expression of DSC2 and repress DSC3 [43]. Although DSC2/desmocollin (r = 0.60) was highly correlated with the NEC gene expression pattern, DSC3 lacked significant correlation. Thus Plakoglobin may signal LEF1 to transcriptionally activate the desmosomal cadherin DSC2 (Figure 14), which functions in epithelial cells as opposed to mesenchymal cells. Plakoglobin also binds and stabilizes NME1/NM23-H1 via CTNNA1/alpha-catenin, which would tend to inhibit metastasis [44], [45] (Figure 14). Plakoglobin binds DSG3/desmoglein (r = 0.38), which prevents entry of plakoglobin into the nucleus [46] (Figure 14). Thus JUP/plakaglobin is central to several epithelial-specific functions shown in the molecular interaction map in Figure 14.

**DSP**/desmoplakin (r = 0.62) binds the epithelia-specific keratin intermediate filaments **KRT8** (r = 0.63), **KRT19** (r = 0.63) and **KRT18** (r = 0.59). In intestinal epithelium, these keratins function to maintain proper architecture of microvilli even without linkage to desmosomes [47]. However in stratified epithelia, keratins appear to be required to maintain desmosomes. During epithelial-mesenchymal transition, disassembly of desmosomes preceded loss of E-cadherin [48]. KRT8/18 reduced the sensitivity of human carcinoma cell lines to cisplatin, and depletion of KRT8/18 increased cisplatin-induced apoptosis [49]. The expression of KRT8 was closely correlated with that of BTG4 (r = 0.54 relative to NEC; r = 0.92 relative to KRT8), which has anti-proliferative properties [50].

### Indirect Interactions with Epithelial Cell Surface Complexes

Several NEC-correlated genes interact indirectly with tight or adherens junctions, regulate their functions, and link them to other cell structures (Figure 14). The largest number of interactions impacting cell surface complexes emerge from transcription factors **GRHL1** and **GRHL2** (r = 0.83 and 0.89), suggesting key rolls in the functions in the of epithelial cell-cell junctions (Figure 14). In particular, GRHL1 and/or 2 up-regulate the transcription of CLDN4 and CDH1, as well as **RAB25** (r = 0.94), whose expression is almost perfectly correlated with the NEC genes. RAB25 also enhances the expression of CLDN4, induces its localization in tight junctions, and activates the transcription of **TACSTD2** (r = 0.62), a binder of CLDN7 at tight junctions [51] (Figure 14). TACSTD2 is expressed in stratified epithelia, but not in colonic or other simple epithelia [26]).

GRHL2 promotes transcription of **ERBB3** (r = 0.54), which forms an oncogenic heterodimer with ERBB2 (r = 0.37) in breast cancer cells [52]. In the NCI-60, ERBB3 was selectively expressed in the 2 epithelial-like breast cancer cell lines (MCF7 and T47D) and in most of the colon lines, but also in most of the melanoma lines. Although GRHL2 is a transcription factor that targets several epithelial-related genes (Figure 14), its alternatively spliced isoform has a dominant-negative effect [52]. GRHL2 also stimulates the expression of PCNA and is generally expressed in carcinomas, but not in normal tissues [53], [54]. This highly NEC-correlated gene, which is also highly correlated with the expression of epithelial-related genes in the CCLE breast, colon, and ovarian cancer cell lines (Figures 10–13), thus may be involved in conferring proliferation capability to epithelial cancer cells, such as would occur in normal epithelial cells during wound healing [55].

The expression of CDH1/E-cadherin is further enhanced by the highly NEC-correlated **LLGL2** (r = 0.80), which associates with tight junctions via the highly NEC-correlated **CRB3** (r = 0.81) (Figure 14). LLGL2 thereby tends to suppress tumor growth and metastasis. In addition, there appears to be an inverse relationship between LLGL2 and SNAI1/snail in regard to transitions between epithelial and mesenchymal cell states [24], [56].

### Interactions in Intestinal Epithelial Cells

Several NEC genes were selectively and sometimes exclusively expressed in the NCI-60 colon cancer cell lines and have intestine-specific functions. Some of their interactions are included in the molecular interaction map in Figure 14.

At the brush border of intestinal epithelial cells, actin fibers are bundled through the action of **VIL1**/villin-1 (r = 0.68) and **PLS1**/plastin-1/fimbrin (r = 0.64) (Figure 14). VIL1 links the actin cytoskeleton to the plasma membrane by binding phosphatidylinositol-4,5-bisphosphate and PLCG1/phospholipase C gamma1 [57] (Figure 14). PLS1 was expressed selectively, and VIL1 exclusively, in the NCI-60 colon cancer cell lines, consistent with their role in crosslinking actin fibers at the core of microvilli in intestinal epithelium.

The normal progression of intestinal epithelial cells from crypt to villus involves the highly NEC-correlated gene **ELMO3** (r = 0.88), which is activated by the transcription factors CDX2 and SP1 [58]. CDX2 is an intestine-specific transcription factor whose expression has been reported to be reduced in most human colon cancer cases. In the NCI-60, it was expressed in only 2 cell lines: colon HCC2998 and HCT15.

**CBLC**/CBL3 (r = 0.86) and **EPHA1** (ephrin receptor A1) (r = 0.82) are both expressed highly selectively in the NEC cell lines, including all of the NCI-60 colon cancer cell lines, except SW620, and both are expressed selectively in the epithelial-like CCLE colon and breast cancer cell lines (not shown). CBLC is expressed specifically in epithelia, most prominently in intestinal epithelia, where it is expressed mainly in post-proliferative cells in villi [59]. Mutation or knockdown of CBLC can promote motility and proliferation of breast and non-small-cell lung cancer cell lines. Moreover, CBLC overexpression can inhibit migration and proliferation in cell culture, as well as tumor growth and metastasis in animals [60].

**EFNA1** (ephrin-A1) (r = 0.53), is a membrane receptor tyrosine kinase that avidly binds its major ligand located on an adjacent cell and is expressed mainly in epithelial tissues. Of the 10 EPHA receptors, only EPHA1 was expressed highly selectively in the NEC cell lines. When activated by interaction with EFNA1, EPHA1 binds and inhibits integrin-linked kinase ILK, enhances adhesion to extracellular matrix, and inhibits cell migration and invasion [61]. Ephrin receptors may stabilize cell-cell or cell-matrix junctions by interacting with CDH1 (E-cadherin), claudins, or integrins [62]. EPHA1 is often overexpressed in colorectal carcinoma. However in advanced disease its expression tends to be low (due to DNA methylation) and associated with lower survival [63].

**FGF19** (r = 0.61) was expressed exclusively in 5 colon cell lines of the NCI-60; its receptor **FGFR4** (r = 0.44) was expressed in most of those colon cancer cell lines but also in the breast cancer MCF7 and several other cell lines. In colon HCT116 and COLO205 (which were among the NCI-60 cell lines that expressed FGF19 and FGFR4), FGF19 promoted tyrosine phosphorylation of CTNNB1/beta-catenin and inhibited its binding to CDH1/E-cadherin [64]. Consequently CTNNB1 became activated and therefore stimulated TCF/LEF transcription factors, leading to enhanced transcription of cyclin D1 and other TCF/LEF-target genes. This pathway from FGF19 to TCF/LEF seems to prime cultured epithelial-like cells for replication. FGF19 uniquely binds and activates FGFR4 [65]. FGF19-activated FGFR4 releases CTNNB1/betaCatenin from CDH1/E-cadherin, allowing CTNNB1 to enter the nucleus and activate cyclin D transcription via TCF/LEF [65] (Figure 14). The above interactions may be relevant to adenopolyposis coli induced by APC mutations, a common pre-cancerous process that activates the same CTNNB1-TCF/LEF pathway.

**MACC1** (metastasis associated colon cancer 1) (r = 0.80) was selectively expressed in the NCI-60 colon cancer cell lines, as well as most of the other NEC cell lines. It binds and activates the MET promoter and enhances cell proliferation, motility and scattering in a MET and HGF-dependent manner that induces nuclear translocation of MACC1. MACC1 and MET mRNA expression are directly correlated with metastasis in colon cancer, and MACC1 is a useful clinical marker for the ability of tumor cells derived from epithelial tissues, such as colon and stomach, to metastasize [66]–[68]. Down-regulation of MACC1 suppressed colorectal cell proliferation, migration and invasion [69]. The MACC1 core promoter has functional sites for AP-1, Sp1, and C/EBP [70], and is a target of miR143, whose expression tends to be diminished in colorectal cancer tissues and cell lines. MACC1 may act as master regulator of the HGF-MET signaling pathway [67], and is strongly expressed in gastric and intestinal epithelia [71].

The selective expression of **CDH17**, as well as CLDN2 and CLDN15, in colon cancer cell lines has already been noted above in relation to Figure 6 (red arrows).

### Regulation of Alternative RNA Splicing

Alterative RNA splicing at consensus nucleotide sequences is an efficient way a master controller protein can simultaneously regulate the function of multiple pathways. Although we do not have adequate data on the expression levels of alternative transcripts, we did find splice-regulators among the highly NEC-correlated (or negatively-correlated) genes.

A central control of mesenchymal to epithelial transition by way of altered RNA splicing involves **ESRP1 and 2** (epithelial splicing regulatory proteins 1 and 2) (r = 0.97, 0.85) [72]. Forced expression of ESRP1 inhibits epithelial-to-mesenchymal transition, and ectopic expression of either ESRP1 or ESRP2 switched RNA splicing of several genes from a mesenchymal to an epithelial pattern. In particular, ESRP1 or 2 switch CTNND1/p120catenin expression to a form that binds CDH1/E-cadherin at adherens junctions [72] (Figure 14).

Fibroblast growth factor **FGF2** (r = –0.44) and its receptor **FGFR1** (r = –0.38) are implicated in repression of epithelial genes via ZEB1/2 [73]. FGFR1 has alternative splice variants that are specific for epithelial and mesenchymal cells, respectively. ESPR1 and 2 mediate a switch to the epithelial variant [73].

mRNA splicing is also modulated by the RNA-binding protein **QKI**/Quaking, which exhibited a negative expression correlation with that of the NEC cell lines (r = –0.71) [74], [75].

### Regulation of Vesicle Transport and Cell Polarity

Vesicles such as endosomes move cell components along microtubules from one site to another, thereby regulating both cell structure and signaling functions at various locations in the cell, and inducing epithelial cell polarity. Internalization and recycling of cell surface components in endosomes regulates signaling and ion channels at the cell surface of polarized cells [76], [77]. Some of the genes implicated in these functions were expressed in the NCI-60 epithelial consensus (NEC) cell lines with high degree of selectivity.

Endocytosis is mediated in part by epsins [78]. Of the 3 epsins, **EPN3**/epsin-3 (r = 0.83) was expressed selectively in the NEC cell lines. EPN3 is involved in the transport of endosomal cargo proteins to and from the apical surface of epithelial cells. These cargo proteins include sodium ion channels, NOTCH ligands, and epithelial growth factor (EGF) receptors, which are removed from the cell surface in endosomes and then may be recycled to the cell surface, thereby regulating the functions of these molecules [78]. The EPN3 N-terminal domain binds PtdIns(3,4) P2, which is enriched at endocytic sites of the plasma membrane; this interaction induces a conformational change in EPN3 that promotes membrane curvature, which promotes the formation of clathrin-coated pits and endocytosis. C-terminal to that domain, EPN3 has a ubiquitin binding-domain that recruits mono- or oligo-ubiquitinated cargo proteins to the nascent endosome. C-terminal to that domain are clathrin-binding sites that recruit the clathrin coat for the endosome. Thus EPN3 is a key component in the production of clathrin-coated endosomes [78].

Epsins are required for intercellular communication mediated by NOTCH ligands and receptors [79]. Of the 4 NOTCH family members, **NOTCH3** (r = 0.46) exhibited significant expression selectivity in the NEC cell lines.

Despite its highly selective expression in the epithelial-like cell lines, however, EPN3 may promote cell migration and invasion [78]; epithelial cells may acquire migratory capability when they become carcinomatous without loosing their epithelial character.

Another factor involved in vesicle transport along microtubules is the small GTPase **RAB25** (r = 0.94), whose expression in the NCI-60 cell lines was almost perfectly selective for the epithelial-like NEC cell lines, and whose expression is prominent in epithelial tissues [80]. The transport vesicles may contain proteins, such as ITGB1 and GLUT1 as cargo for delivery to certain locations in the cell [81]. RAB25 maintains epithelial cell polarity by transporting cargo-containing vesicles to the apical or lateral surfaces of cells in an epithelium or to the focal adhesion processes of epithelial cells that have undergone a transition to mesenchymal form [80]. The expression of RAB25 is suppressed by the mesenchymal marker **VIM**/vimentin (r = –0.86), but is activated by **GRHL2** (grainyhead-like 2) (r = 0.89) [82] (Figure 14).

Vesicle transport is promoted by ARF6, which is inactivated by the GTPase activating protein (GAP) **ADAP1**/CENTA1/centaurin-alpha1 (r = 0.82). In its active GTP form, ARF6 promotes clathrin-dependent endocytosis of CDH1/E-cadherin with consequent disassembly of adherens junctions and conferring migration and invasion capabilities [34], [35]. ADAP1 inhibits these actions, thereby tending to maintain epithelial cell-cell junctions, consistent with the highly selective expression in NEC cell lines (Figure 14). ADAP1 also regulates cargo transport between plasma membrane and vesicles, and regulates cortical actin cytoskeleton for several processes, including focal adhesions [83].

Motor function for vesicle transport is provided by **MYO5B** (r = 0.79) and **MYO5C** (r = 0.73). MYO5B is ubiquitously expressed, whereas MYO5C is expressed mainly in glandular and epithelial cells [84]. However both proteins were selectively expressed in the NEC cell lines, suggesting specific function in epithelial-like cells. The vesicle transport function of MYO5B may be coordinated by complex formation with **RAB25** and/or **RAB11A** (r = 0.49) [85] (Figure 14).

**PLEKHG6**/MyoGEF (r = 0.85) is recruited by ezrin to the apical pole of epithelial cells, where it induces the formation of microvilli and membrane ruffles. It forms a complex with ezrin and RhoG or with ezrin and ELMO (an effector of RhoG), wherein PLEKHG6 can activate RhoG in response to EGF in EGFR-expressing cells [86]. PLEKHG6 and ezrin are both required for macropinocytosis [87]. PLEKHG6 protein can also bind GIPC1 and is implicated in cancer cell polarization and invasion [88]. PLEKHG6 is a target of miR-let7i [89]. Thus PLEKHG6, RhoG, ezrin, ELMO, GIPC1, and miR-let7i may be part of a network or networks that regulate cell surface morphology and function in epithelial tumor cells.

**Figure 15:**
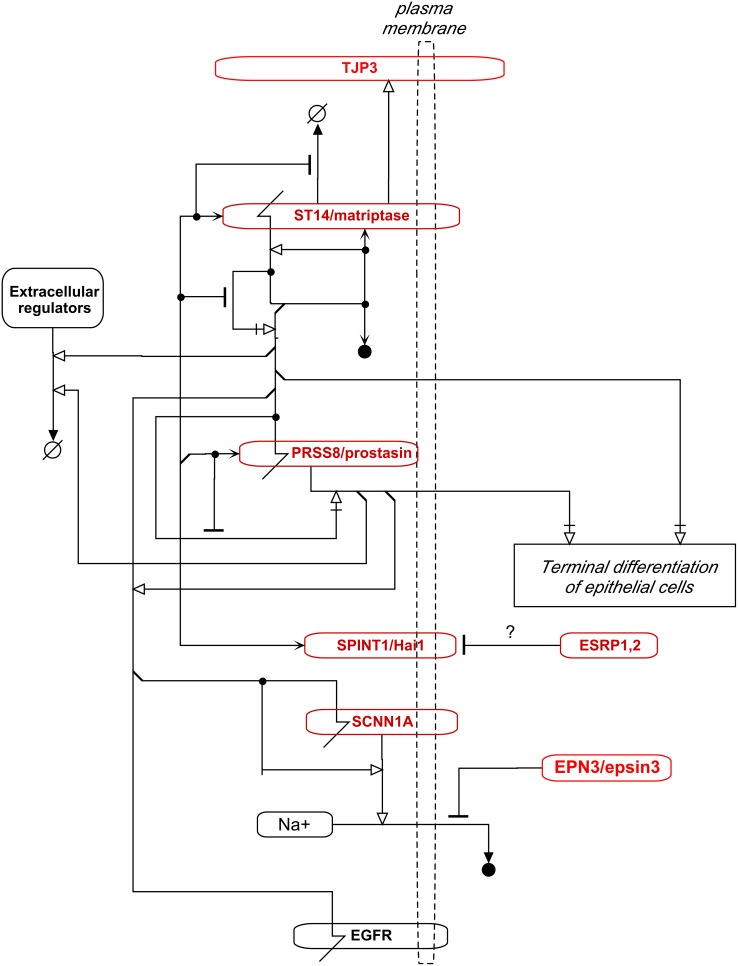
Regulation of the balance between proliferative and terminally differentiating epithelial cells, based on descriptions by [105], [107]–[109]}[106]. The genes that were expressed selectively in the NEC cell lines are denoted in red. Terminal differentiation of epithelial cells requires IRF6, NOTCH1, ST14/matriptase, and PRSS8/prostasin. The actions of the latter two are inhibited by SPINT1/hai1. The continued proliferation or cell division on the path to terminal differentiation requires MYC and MYB. IRF6 and MYB transcriptionally activate OVOL1, which down-regulates the transcription of OVOL2, MYB, and MYC, and tends to inhibit terminal differentiation. OVOL2 inhibits the transcription of MYC and NOTCH1. TP63 enhances the transcription of IRF6, but is down-regulated by IRF6. (See text for further description of the model).

**SCNN1A**/ENaC (r = 0.83) is a subunit of an epithelial sodium channel that facilitates electrogenic uptake of sodium from the luminal surface of epithelia. It is activated by cleavage of its extracellular domain by **ST14**/matriptase (r = 0.89) [90] (Figure 15). These sodium channels are removed from the plasma membrane in endosomes formed with the participation of clathrin and **EPN3** (r = 0.83) and may be recycled. Thus at least 3 genes involved in the regulation of these channels were expressed highly selectively in the NEC cell lines.

In summary, several of the epithelial-specific genes, identified by their selective expression in the NEC cell lines, are implicated in endocytosis, vesicle transport and cell polarity, including **EPN3**/epsin-3 (r = 0.83), **RAB25** (r = 0.94), **GRHL2** (r = 0.89), **ADAP1**/CENTA1 (r = 0.82), **MYO5B** (r = 0.79), **MYO5C** (r = 0.73), **AP1M2** (r = 0.75), and **PLEKHG6**/MyoGEF (r = 0.85).

### Genes Involved in Ca(2+) Signaling

Ca(2+) signaling is involved in coordinating the transport of cargo-containing vesicles from endoplasmic reticulum to Golgi to plasma membrane [91]; this process may be involved in transport of cargo to specific regions of plasma membrane to maintain epithelial cell polarity. Three Ca(2+)-related genes exhibited highly selective expression in the NEC cell lines: **ATP2C2**/SPCA2 (r = 0.90), **S100A14** (r = 0.89), and **BSPRY** (B-box and SPRY domain containing) (r = 0.87). The extraordinary expression selectivity of these genes in the NEC cell lines suggest that their Ca(2+)-regulated gene products have specific roles in epithelial tumor cell lines, which however are still not well understood.

**ATP2C2/SPCA2** is a Ca(2+)-ATPase that activates calcium channel ORAI1 and mediates uptake of Ca(2+) and Mn(2+) by Golgi membranes; deficiency leads to increased cytosolic Ca(2+) and Golgi disruption [92]. ATP2C2 has a Golgi-like distribution in the cell and is abundant in colon, lung, and lactating breast. It localizes at the plasma membrane of intestinal epithelial cells; in epidermal cells however it has a perinuclear localization consistent with localization at sites of calcium storage [93]–[95]. ATP2C2 is transcriptionally activated by **BHLHA15**/MIST1 (r = 0.51) in serous exocrine epithelial cells, including acini of pancreas, salivary, and lactating mammary glands [94].

**S100A14**, an EF-hand Ca(2+)-binding protein, is the only S100A-family member whose expression was highly selective for the NEC cell lines. It is expressed in several normal epithelial tissues, and low expression is associated with poor prognosis in colorectal tumors and adenocarcinomas of the small intestine [96], [97]. It is markedly overexpressed in some tumors of ovary, breast and uterus, but down-regulated in several other tumor tissues. It promotes or inhibits tumor cell invasiveness, depending on p53 status. Promotion of cell invasiveness by S100A14 is mediated by induction of metalloproteinase MMP2, which is transcriptionally repressed by p53, which however is in turn transcriptionally repressed by S100A14 [98]. Although the details of these interactions remain to be worked out, our finding suggest that they are specific for epithelial cancer cells.

**BSPRY** co-localizes and negatively regulates TRPV5, which is a channel for active calcium uptake by epithelia. BSPRY inhibited the TRPV5-mediated influx of Ca(2+) in kidney cells. Thus BSPRY may be a negative regulator of Ca(2+) transport [99]. Its role in epithelial tumor cells, suggested by its high selectivity of expression in the NEC cell lines, however remains to be further elucidated.

### Regulators of Terminal Differentiation of Epithelial Cells

**Figure 16:**
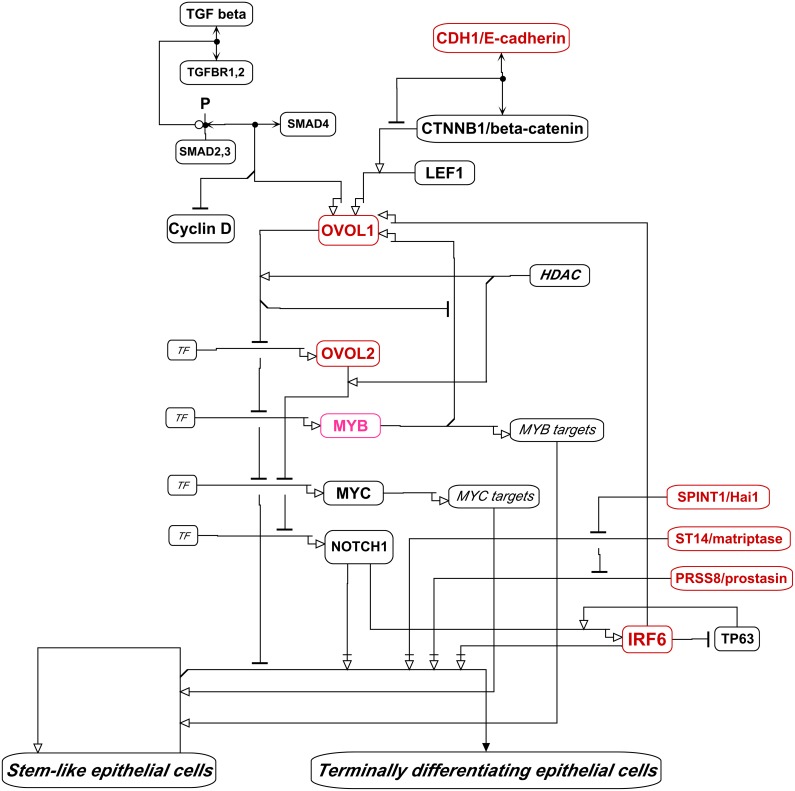
Regulation of the balance between proliferative and terminally differentiating epithelial cells, based on descriptions by [105], [107]–[109]}[106]. The interaction system involving ST14/matriptase, PRSS8/prostasin, and SPINT1/Hai1 (detailed in Figure 15) is shown here in abbreviated form. The genes that were expressed selectively in the NEC cell lines are denoted in red. The diagram shows terminal differentiation of epithelial cells requiring IRF6, NOTCH1, ST14/matriptase, and PRSS8/prostasin. The actions of the latter two are inhibited by SPINT1/hai1. The continued proliferation or cell division on the path to terminal differentiation requires MYC and MYB. IRF6 and MYB transcriptionally activate OVOL1, which down-regulates the transcription of OVOL2, MYB, and MYC, and tends to inhibit terminal differentiation. OVOL2 inhibits the transcription of MYC and NOTCH1. TP63 enhances the transcription of IRF6, but is down-regulated by IRF6. Also shown is the regulation of OVOL1 by the TGF beta and beta-catenin pathways.

The maintenance and cell renewal in epithelial tissues requires delicate controls on cell proliferation and terminal differentiation [100]. These processes, particularly in skin and kidney, are controlled in part by **IRF6** (r = 0.93), **OVOL1** (r = 0.83) and **OVOL2** (r = 0.75) (Figure 16). Terminal differentiation of epithelial cells is also delicately regulated by extracellular serine proteases **ST14**/matriptase (r = 0.89) and **PRSS8**/prostasin/CAP1 (r = 0.83), and their inhibitors **SPINT1**/Hai1 (r = 0.88) and **SPINT2**/Hai2 (r = 0.66). All 7 of these terminal differentiation regulators were expressed highly selectively in the NCI-60 epithelial consensus (NEC) cell lines. This is consistent with a continued function of terminal differentiation controls in epithelial cancer cell lines. The known interactions regulating epithelial cell proliferation and differentiation by these components is depicted as molecular interaction maps in Figures 15 and 16, which show how these genes modulate the balance between proliferation and differentiation of epithelial stem cells.

ST14/matriptase and PRSS8/prostasin are required for terminal differentiation of epithelia [101]. Together with their inhibitor, SPINT1/Hai1, they maintain the structural and functional integrity of epithelia [102], [103]. All 3 proteins are usually co-localized in epithelial cells [103], [104].

**IRF6** (r = 0.93) drives the differentiation of epithelial cells, although the mechanism has not been fully defined; down-regulation of IRF6 inhibits keratinocyte differentiation and promotes RAS-induced tumorigenesis [105], [106]. IRF6 transcriptionally targets OVOL1, which is an inhibitor of MYC [107]. OVOL1 and OVOL2 down-regulate proliferation of both stem cells and proliferating cells through transcriptional inhibition of MYC and **MYB** (r = 0.35) [108], [109] (Figures 14 and 16). OVOL1 is also transcriptionally activated by the CTNNB1-LEF1 and TGFBR-SMAD pathways, which may thereby control excessive cell proliferation (Figure 16). As well as inhibiting the transcription of the MYB gene, OVOL1 competes with MYB for binding to promoters of MYB-target genes, including the OVOL1 gene itself. OVOL1 protein thus may bind its own promoter and inhibit its own transcription. OVOL2 is expressed in basal layer of epidermis and is thought to be associated with epidermal stem cells. It directly represses MYC and NOTCH1, the latter being required for terminal differentiation of squamous epithelia. (NOTCH1 was expressed in all NCI-60 cell lines, and therefore did not correlate with NEC cell lines.) In a negative feedback loop, IRF6 transcription is enhanced by TP63 (in conjunction with NOTCH1) and induces the proteasomal degradation of delta-N-TP63 [110]. In summary, OVOL1 and 2 are expressed in a variety of epithelial tissues where they seem to restrain cell proliferation and terminal differentiation, while retaining the long-term proliferative potential of stem cells. They seem to modulate the balance between proliferation and differentiation of epithelial stem cells [108], [109]. Although the intricacies of the matriptase-prostasin-Hai1 protease system have not been fully elucidated, the essentials of the core of the system have been proposed [101], [102] and are part of the basis of the molecular interaction maps in Figures 15 and 16.

**ST14**/Matriptase (r = 0.89) is thought to be auto-activated by cleavage in trans after oligomerization [102], [103]. Nascent ST14/matriptase appears to be stabilized by tight binding of **SPINT1**/Hai1 (r = 0.88), which inhibits ST14/matriptase enzyme activity. SPINT1/Hai1 is an unusually effective, albeit reversible, and rapidly acting inhibitor [102], [111]. SPINT1/Hai1 is often present in large (e.g., ∼10-fold) excess over ST14/matriptase, which is consistent with rapid inhibition of matriptase. This arrangement may cause matriptase activity to be short-lived after transient activation [102], [103], which could serve to clear the cell’s interaction and signaling environment to allow new environmental factors to be sensed. ST14/matriptase also activates **PRSS8**/prostasin (r = 0.83), a glycosylphosphatidylinositol-anchored serine protease on the plasma membrane. Activation of prostasin and matriptase occurs by cleavage of these proteins, which are both rapidly inhibited by binding SPINT1/Hai1 [102], [111] (Figures 15 and 16). Matriptase and prostasin are commonly co-expressed in breast cancer cell lines [112]; in the NCI-60, they were co-expressed along with SPINT1/Hai1 in the 2 breast cancer cell lines of the epithelial consensus. Matriptase-deficient and prostasin-deficient mice have nearly identical epidermal defects [102]. Matriptase cleaves and activates prostasin during the brief time before being inhibited by Hai1 [113]; indeed SPINT1/Hai1 rapidly inhibits both matriptase and prostasin [102]. Thus transiently activated matriptase and prostasin, both rapidly inactivated by SPINT1/Hai1, may work together to cleave and activate or inactivate epithelia regulatory proteins so as to keep up with potentially changing cell environment. When matriptase activity is uncontrolled, it may destroy or over-activate normal signaling elements in the pericellular environment, leading to uncontrolled growth in carcinomas.

**PRSS8**/prostasin (r = 0.83) is abundantly expressed in normal breast and prostate tissues, but down-regulated in cancers. Matriptase can cleave the extracellular domain of EGFR, and prostasin can enhance this action [101]. EGFR, like matriptase, localizes to the basolateral surfaces of epithelial cells. Thus EGFR-induced signaling may be one of the pericellular regulatory processes that is periodically cleared by the matriptase-prostasin-Hai1 system in epithelial cells. If periodic activation of prostasin fails, epithelial cells may become permissive for EGFR-induced transition to a migratory of mesenchymal form.

**SCNN1A**/ENaC (r = 0.83) is another protein that is cleavage-activated by ST14/matriptase and has already been discussed above.

**EHF**/ESE3 (r = 0.80) regulates the self-renewal of stem-like epithelial cell and favors transition to a differentiation pathway, at least in prostate cells [114]. EHF/ESE3 inhibits the transcription of mesenchymal effector genes, such as ZEB2 and TWIST1 and consequently inhibits epithelial-to-mesenchmal transition. Suppression of EHF/ESE3 favors transition to malignancy. EHF/ESE3 is suppressed by mesenchymal gene VIM/vimentin [82].

**ELL3** (r = 0.75) promotes proliferation and cancer stem cell-like properties of breast cancer cells and may confer resistance to 5-fluorouracil; these actions are largely due to activation of MEK/ERK signaling [115]. ELL3 is a transcription elongation factor that suppresses the transient pausing of RNA polymerase II. Additionally, ELL3 stimulates epithelial-mesenchymal transition, suppresses TP53, and promotes pluripotent differentiation of embryonic stem cells while inhibiting apoptosis [116]. ELL3 activates the transcription of ZEB1, which is surprising, because ELL3 and ZEB1 exhibit inverse expression in the NCI-60 (as well as in the CCLE breast cancer cell lines). ELL3 was selectively and nearly exclusively expressed in the 2 breast and 7 colon cancer cell lines of the NCI-60 (NEC) epithelial profile. In the CCLE data, ELL3 expression was highly correlated with the NEC epithelial profile, particularly in the CCLE breast cancer cell lines; ELL3 was highly negatively correlated with ZEB1/2 (data not shown).

### Innate Immune Pathways in Epithelial Cells

**IL17RE** (r = 0.81), in complex with IL17RA, binds IL17C and engages in an autocrine mechanism to induce innate immune pathways in epithelial cells [117].

### Genes Negatively Correlated with the NEC Pattern

In addition to positively NEC-correlated genes (Tables 2 and 3), we found many negatively correlated genes, including genes such as VIM/vimentin, associated with mesenchymal cell character (Table 4, Figure 3). A clustered image map (CIM) of NCI-60 gene expression, comparing the genes most highly positive with those most negative relative to the NEC pattern showed a sharp distinction between epithelial and non-epithelial genes (Figure 10). As expected, there is a distinct cluster of NEC cell lines at the top of the CIM. The other cell lines showed consistently low expression of the positively correlated “epithelial” genes. This demonstrates the sharp distinction between epithelial and non-epithelial gene expression in the NCI-60 cell lines and was confirmed in the CCLE breast, colon, and ovarian cancer cell lines (Figures 11–13).

### Overview and Conclusions

The large amount of accumulated information on cancer-related molecular interactions and gene expression patterns challenges us to use that information to comprehend cell functions. The current work used gene expression data to elucidate cancer cell regulatory functions, based on the premise that genes expressed together in a variety of cancer cell types are likely to function together. We showed how genes mutually co-expressed in human tumor cell lines comprised molecular interaction networks regulating coherent functions in epithelial-like cells. The focus was on genes that confer epithelial-related functions and serves as a foundation for subsequent investigation of the mechanisms that control transitions between epithelial and mesenchymal phenotype.

A unique aspect of the current work is the assembly of detailed and comprehensive molecular interaction maps (MIMs) of networks regulating structure and function of epithelial cancer cells (Figures 14–16). That level of detail and coverage will help in the design and interpretation of experiments in which particular genes or interactions are stimulated or inhibited, including the potential effects and side-effects of pharmacological intervention.

We first identified a set of mutually co-expressed tight-junction and cadherin family genes in a subset of NCI-60 human tumor cell lines derived from epithelial tissues (“NCI-60 epithelial consensus, NEC” genes and cell lines) (Table 1, Figure 4). Based on selective expression by these NEC cell lines, we derived 76 genes, among which 44 had published information relating to epithelial functions (Table 2). For many of the 32 remaining genes, there was little or no published information (Table 3), but their co-expressions suggest that many of them may have epithelial-related functions that remain to be discovered. The epithelial signature for tumor cell lines suggested by the NEC genes was supported by data from the CCLE human breast and colon cell lines (Figures 5 and 6). Moreover, the dichotomy between epithelial and non-epithelial/mesenchymal gene expression is clear in clustered image maps (CIMs) of NCI-60 as well as CCLE breast, colon, and ovarian cancer cell lines (Figures 10–13). The same was true for CCLE pancreas and stomach cancer cell lines (not shown). A CIM of the large number of CCLE lung cancer cell lines showed distinct epithelial and non-epithelial/mesenchymal gene expression patterns, although some cell lines showed mixed patterns arising perhaps from mixtures of cell types in those lines (not shown).

We systematically assembled the recently published molecular interaction data for the highly NEC-correlated genes in Table 2, as well as for some of the lesser correlated genes, and organized them, in molecular interaction maps (Figures 14–16).

It is remarkable how many NEC-correlated genes were found to participate in a network of interactions at cell-cell junctions (Figure 14; NEC-correlated genes are shown in red). The NEC-correlated genes in the MIM include 7 tight junction genes, 3 adherens junction genes, 6 desmosomal genes, and 17 genes whose products interact directly with the tight junction, adherens junction, and/or desmosome structures. Among these, the most highly connected in the network is the GRHL1/2 (r = 0.83, 0.89) pair of transcription factors, which target at least 5 NEC genes or their directly interacting species. Also highly connected is JUP/plakoglobin, which interacts with components of adherens junctions and desmosomes, as well as transcription factors TCF/LEF. Its positive effect on TCF/LEF and competition with CTNNB1/beta-catenin for binding to CDH1/E-cadherin (which would tend to release beta-catenin and allow it to enter the nucleus) suggests that plakoglobin favors cell proliferation. These interactions may feed into the pathway where mutations of the APC gene, common in pre-cancerous colonic polyps, abrogate the degradation of CTNNB1.

NEC-correlated genes may link between cell-cell junctions and cytoskeleton: CGN/cingulin (r = 0.80) can bind both tight junctions and F-actin; CAMSAP3 (r = 0.76) links adherens junctions to microtubules; DSP/desmoplakin (r = 0.62) links desmosomes to the intermediate filament keratins KRT8, 18, and 19 (r = 0.63, 0.63, 0.59) (Figure 14).

An unexpected finding was the disparity between expression of ZEB1 and ZEB2, which seems not to have been reported previously. In the NCI-60 melanoma cell lines, ZEB2 had relatively high expression, whereas expression of ZEB1 was relatively low (Figure 10). In the CCLE breast, colon, and ovarian cancer cell lines, on the other hand, the converse was observed (Figures 11–13).

Many NEC-correlated genes have functions involved in maintaining apical-basal polarity, a key feature of epithelial cells [24] (Figure 14). Prominent among those genes are LLGL2 (r = 0.80), CRB3 (r = 0.81), INADL (r = 0.69), VIL1/villin (r = 0.68), and PLS1/fimbrin (r = 0.64). A particularly important process required to maintain apical-basal polarity is the control of vesicular transport of specific molecules to apical versus basal locations in the cell. Involved in this function were the highly NEC-correlated genes EPN3/epsin-3 (r = 0.83), RAB25 (r = 0.94), GRHL2 (r = 0.89), ADAP1 (r = 0.82), and AP1M2 (r = 0.75). These genes are implicated in the incorporation of cell surface components into vesicles. In addition, the motor proteins MYO5B (r = 0.79) and MYO5C (r = 0.73) move cargo-containing vesicles along cytoskeletal tracks Kashyap et al., 2013). Epithelial cell-cell junction components, such as CDH1/E-cadherin (r = 0.77), can be removed by clathrin-dependent endocytosis, leading to degradation and consequent disassembly of adherens junctions. This process is inhibited ADAP1 (Figure 14), which thereby helps maintain epithelial cell-cell junctions. Also related to this process is the highly NEC-correlated CNN1A/ENaC (r = 0.83), which is involved in regulation of ion channels in the plasma membrane [90] (Figure 15).

An efficient mechanism of switching between epithelial and mesenchymal phenotypes may be by way of alternative RNA splicing induced by the highly NEC-correlated genes ESRP1 and ESRP2 (r = 0.97 and 0.85, respectively). ESRP1 had the strongest and essentially perfect correlation with selective expression in NEC cell lines; this is striking because it was not one of the input genes from which the consensus cell lines were derived, nor is it related to any of those genes by sequence, function, or chromosome location. ESRP1 and 2 are not tight junction or adherens junction genes and do not interact with those structures. Instead they control mRNA splicing and are master regulators of an epithelial splicing network [72].

Cancers derived from epithelia are often defective in the normal transition of cells having sustained proliferative potential to cells undergoing terminal differentiation. Since defective regulation of terminal differentiation allows uncontrolled proliferation of the tumor cells, it is important to understand how terminal differentiation is normally regulated and how the regulation is defective in tumors. Indeed some investigated therapies attempt to induce terminal differentiation of epithelial, as well as other tumor types. We found that a major part of the network regulating epithelial terminal differentiation involves highly NEC-selective genes (Table 2), which helped us assemble a network model of the differentiation control system. The molecular interaction maps in Figures 15 and 16, which are based largely on highly NEC-selective genes (depicted in red), propose the following model for a two-part system that intricately regulates the terminal differentiation of epithelia. The interactions of each gene/protein shown in Figures 15 and 16 were summarized in greater detail in the Results section, together with supporting references.

Epithelial terminal differentiation requires a cleaved form of PRSS8/prostasin (r = 0.83), the production of which depends in turn on a cleaved form of ST14/matriptase (r = 0.89) (Figure 15). The protease responsible for these cleavage reactions, as well as of those mentioned below and indicated in Figure 15, is in fact a dimeric form of ST14/matriptase. The active cleaved forms would accumulate and reach a balance with degradation processes, producing a long-term integration of the initiating signals over time. The stimulatory effects of PRSS8/prostasin and ST14/matriptase are negatively regulated by SPINT1/Hai1 (r = 0.88). All 3 of these genes were expressed highly selectively in the NEC cell lines (z-score correlations 0.83, 0.89, 0.88, respectively; Table 2). The production of the active cleavage products take place in the extracellular region near the cell surface. By diffusion, the differentiation stimulus would therefore be shared among neighboring cells. Thus the differentiation stimulus would spread among the cells of the epithelium, providing an coherent differentiation rate among the cells. If the negative regulation component of the system is defective or does not spread adequately, some regions of the tissue could proliferate without adequate control, as occurs in tumors.

In a second level of regulation, epithelial terminal differentiation is dependent on IRF6 (r = 0.93) and negatively regulated by OVOL1 and OVOL2 (Figure 16). IRF6, OVOL1, and OVOL2 are highly NEC-correlated and seem to be the core of this part of the regulation system, which appears to be further regulated by an intricate arrangement of positive and negative feedback loops (Figures 15 and 16). It seems that NOTCH1 is stimulated by IRF6 and negatively regulated by OVOL2, which is itself negatively regulated by OVOL1. OVOL1 is transcriptionally activated by IRF6, which is itself transcriptionally activated by NOTCH1. Thus IRF6 stimulates epithelial terminal differentiation, but this effect may be dampened in a time-limited fashion by the transcriptional stimulation of OVOL1.

Inputs to the system via regulation of OVOL1 can occur via the TGF beta and beta-catenin pathways. Both pathways transcriptionally activate OVOL1, thereby restricting epithelial cell proliferation and differentiation. The TGF beta pathway couples that action with inhibition of cell proliferation via regulation of cyclin D. The transcriptional inhibition of OVOL1 via beta-catenin is dampened by binding to CDH1/E-cadherin.

Inputs to the system via regulation of OVOL1 can occur via the TGF beta and beta-catenin pathways. Both pathways transcriptionally activate OVOL1, thereby restricting epithelial cell proliferation and differentiation. The TGF beta pathway couples that action with inhibition of cell proliferation via regulation of cyclin D. The transcriptional inhibition of OVOL1 via beta-catenin is dampened by binding to CDH1/E-cadherin.

This model suggests how epithelial terminal differentiation may be highly integrated, both in time and over the cell population, and how some inputs regulate the system. These conjectures invite computer simulations and cell culture experiments and may provide deeper understanding of the regulation defects in cancer.

The unusually high NEC correlations of IRF6 (r = 0.93), OVOL1 (r = 0.83), OVOL2 (r = 0.75), ST14/matriptase (r = 0.89), PRSS8/prostasin (r = 0.83), and SPINT1/Hai1 (r = 0.88) are striking and indicate that these proliferation/differentiation control genes work together specifically in epithelial tumor cell lines.

Finally, it may be noted that expression of many of these NEC-correlated genes is associated with relatively low carcinoma aggressiveness. On the other hand, many of the NEC negatively correlated genes appear to be involved in functions associated with non-epithelial or mesenchymal cell types. These genes and their functions in transitions between epithelial and mesenchymal phenotypes are of great interest to cancer biology.

In summary, gene expression correlations at the mRNA level were remarkably successful as a means to delineate network functions in epithelial-like human tumor cell lines. The expression of a subset of tight-junction genes provided a key whereby expression correlation identified genes functioning in a broad range of epithelia-related molecular interactions. The gene expression correlations, initially derived in the NCI-60 human tumor cell lines, were consistent with gene expression patterns in the much larger set of CCLE human tumor cell lines of epithelial origin, which provided additional information. The mutually correlated expression of a set of tight junction genes provided a signature for epithelial-like cancer cell lines, distinct from mesenchymal-like tumor cells. The retrieved tight-junction expression-correlated genes had a major role in molecular interaction maps depicting many functions at epithelial cell-cell junctions and depicting regulation of the balance between proliferative and terminally differentiating epithelial cells. Our studies implicated tight-junction expression-correlated genes in a variety of structural and functional characteristics of epithelial cells: (1) maintenance of junction complexes and desmosomes; (2) maintenance of apical-basal polarity by vesicle transport of appropriate components to apical or basal regions of the cell; (3) linkage of cell-cell junction components to actin, microtubule, and intermediate filament cytoskeletons; (4) control of endosomal recycling or degradation of cell-cell junction components; (5) formation of microvilli in intestinal epithelial cells; (6) regulation of RNA splicing in a manner promoting epithelial gene variants; (7) regulation of terminal differentiation of epithelial cells; (8) suppression of tumor growth and metastasis; and (9) inhibition of the epithelial-to-mesenchymal transition as well as promotion of the reverse transition.
